# Supplementary material for: A global genomic analysis of Salmonella Concord reveals lineages with high antimicrobial resistance in Ethiopia
Source: Nat Commun. 2023 Jun 14;14:3517. doi: 10.1038/s41467-023-38902-x (PMC10267216; doi:10.1038/s41467-023-38902-x)
Supplement: Supplementary file 1 — Supplementary information [file 41467_2023_38902_MOESM1_ESM.pdf]

Supplementary Tables and Figures

A global genomic analysis of *Salmonella* Concord reveals lineages with high antimicrobial resistance in Ethiopia

Wim L. Cuypers[1,2*], Pieter Meysman[1], François-Xavier Weill[3], Rene S. Hendriksen[4], Getenet Beyene[5], John Wain[6,7], Satheesh Nair[8], Marie A. Chattaway[8], Blanca M. Perez-Sepulveda[9], Pieter-Jan Ceyssens[10], Tessa de Block[11], Winnie W. Y.Lee[8,12], Maria Pardos de la Gandara[3], Christian Kornschober[13], Jacob Moran-Gilad[14], Kees Veldman[15], Martin Cormican[16], Mia Torpdahl[17], Patricia I. Fields[18], Tomáš Černý[19], Liselotte Hardy[2], Bieke Tack[2,20], Kate C. Mellor[21,22], Nicholas Thomson[21,22], Gordon Dougan[23], Stijn Deborggraeve[24], Jan Jacobs[2,20], Kris Laukens[1], Sandra Van Puyvelde[22, 23, 25*]

[1]Adrem Data Lab, Department of Computer Science, University of Antwerp, Antwerp, Belgium

[2]Tropical Bacteriology Unit, Department of Clinical Sciences, Institute of Tropical Medicine, Antwerp, Belgium

[3]Institut Pasteur, Université Paris Cité, Unité des bactéries pathogènes entériques, F-75015, Paris, France

[4]Technical University of Denmark, National Food Institute (DTU-Food), Research Group of Global Capacity Building, Kgs., Lyngby, Denmark

[5]Department of Medical Laboratory Sciences, Faculty of Health Sciences, Jimma University, Jimma, Ethiopia

[6]Quadram Institute Bioscience, Norwich Research Park, Norwich, UK

[7]Norwich Medical School, University of East Anglia, Norwich, UK

[8]Gastrointestinal Bacterial Reference Unit, United Kingdom Health Security Agency, Colindale, London, UK

[9]Institute of Infection, Veterinary & Ecological Sciences, University of Liverpool, Liverpool, UK

[10]Division of Human Bacterial Diseases, Sciensano, Brussels, Belgium

[11]Clinical Reference Laboratory, Department of Clinical Sciences, Institute of Tropical Medicine, Antwerp, Belgium

[12]MRC Centre for Molecular Bacteriology and Infection, Imperial College London, London, UK

[13]Austrian Agency for Health and Food Safety (AGES), Institute for Medical Microbiology and Hygiene, 8010 Graz, Austria

[14]Department of Health Policy and Management, School of Public Health, Faculty of Health Sciences, Ben Gurion University of the Negev, Israel

[15]Department of Bacteriology, Host Pathogen Interaction & Diagnostics, Wageningen Bioveterinary Research, Lelystad, The Netherlands

[16]Antimicrobial Resistance and Microbial Ecology Group, School of Medicine, University of Galway, Galway, Ireland

[17]Department of Bacteriology, Mycology & Parasitology, Statens Serum Institut, 5 Artillerivej, DK-2300 Copenhagen S, Denmark

[18]Division of Foodborne, Waterborne and Environmental Diseases, Centers for Disease Control and Prevention, Atlanta, Georgia, USA

[19]National Reference Laboratory for salmonella, State Veterinary Institute Prague, Prague, Czech Republic

[20]Department of Microbiology, Immunology and Transplantation, KU Leuven, Leuven, Belgium

[21]London School of Hygiene and Tropical Medicine, Bloomsbury, London, UK

[22]Wellcome Trust Sanger Institute, Genome Campus, Hinxton, Cambridge, United Kingdom

[23]Cambridge Institute of Therapeutic Immunology & Infectious Disease (CITIID),Department of Medicine, University of Cambridge, Cambridge CB2 0SP, United Kingdom

[24]Department of Biomedical Sciences, Institute of Tropical Medicine, Antwerp, Belgium

[25]Laboratory of Medical Microbiology, Vaccine & Infectious Disease Institute, University of Antwerp, Antwerp, Belgium

*Correspondence should be addressed to W.L.C and S.V.P (email: wim.cuypers@uantwerpen.be and sandra.VanPuyvelde@uantwerpen.be)

Table of contents

Supplementary Figures 3

Supplementary Figure 1: S. Concord super-lineage B (HC2000_141) 4

Supplementary Figure 2: *S*. Concord super-lineage C (HC2000_177997) 5

Supplementary Figure 3: Detailed view of *S*. Concord super-lineage A lineages 1 (L1) and 2 (L2) 7

Supplementary Figure 4: Invasiveness index predicted for all *S*. Concord Super-lineage A isolates based on genomic features 8

Supplementary Figure 5: Detail of super-lineage A lineage 8 (L8) showing the mixed occurrence of recent and historical isolates 10

Supplementary Figure 6: Pairwise SNP differences within each *S*. Concord Super-lineage A lineage 11

Supplementary Figure 7: Detail of super-lineage A lineage 6 (L6) showing the close relatedness of isolates from Tahini isolated in Israel and clinical isolates 13

Supplementary Figure 8: Close relatedness of clinical isolates and isolates from Halva food products from Turkey in super-lineage A lineage 7 (L7) 15

Supplementary Figure 9: Complete set of AMR genes and plasmid replicon genes identified in *S*. Concord draft genomes, including AMR genes that do not contribute to the MDR, XDR and PDR phenotypes and less common replicon types 17

Supplementary Figure 10: Genomic AMR combinations in *S*. Concord super-lineage A 19

Supplementary Figure 11: Chromosomal AMR cassettes flanked by IS*1R* were integrated at different positions in the long read sequenced *S.* Concord isolates and varied in length 21

Supplementary Figure 12: Chromosomal AMR cassettes flanked by IS1R varied in composition in lineages 3 and 4 23

Supplementary Figure 13: IncHI2 plasmids and a chromosomally integrated IncHI2 plasmid shared common backbone elements and differed in regions encoding AMR 24

Supplementary Figure 14: The XDR hybrid plasmid identified in isolate 32640_1_296 (middle track; part of Lineage 5) reference genome contained backbone elements derived from IncHI2 and IncA/C plasmids 26

Supplementary Figure 15: Association of AMR genotype and phenotype for first- and second-line antimicrobials recommended to treat invasive *Salmonella* infections 28

Supplementary Figure 16: Association of AMR genotype and phenotype for alternative antimicrobials 30

Supplementary Tables 31

Supplementary Table 1: Presence-absence variations in genes linked to increased invasive potential in L4 isolates. 32

Supplementary Table 2: Comparison of genotypic and phenotypic resistance or susceptibility for antimicrobials that can be used to treat invasive *Salmonella* infections. 33

Supplementary Table 3: Assembly summary of long read sequenced genomes. 34

References 36

Supplementary Figures

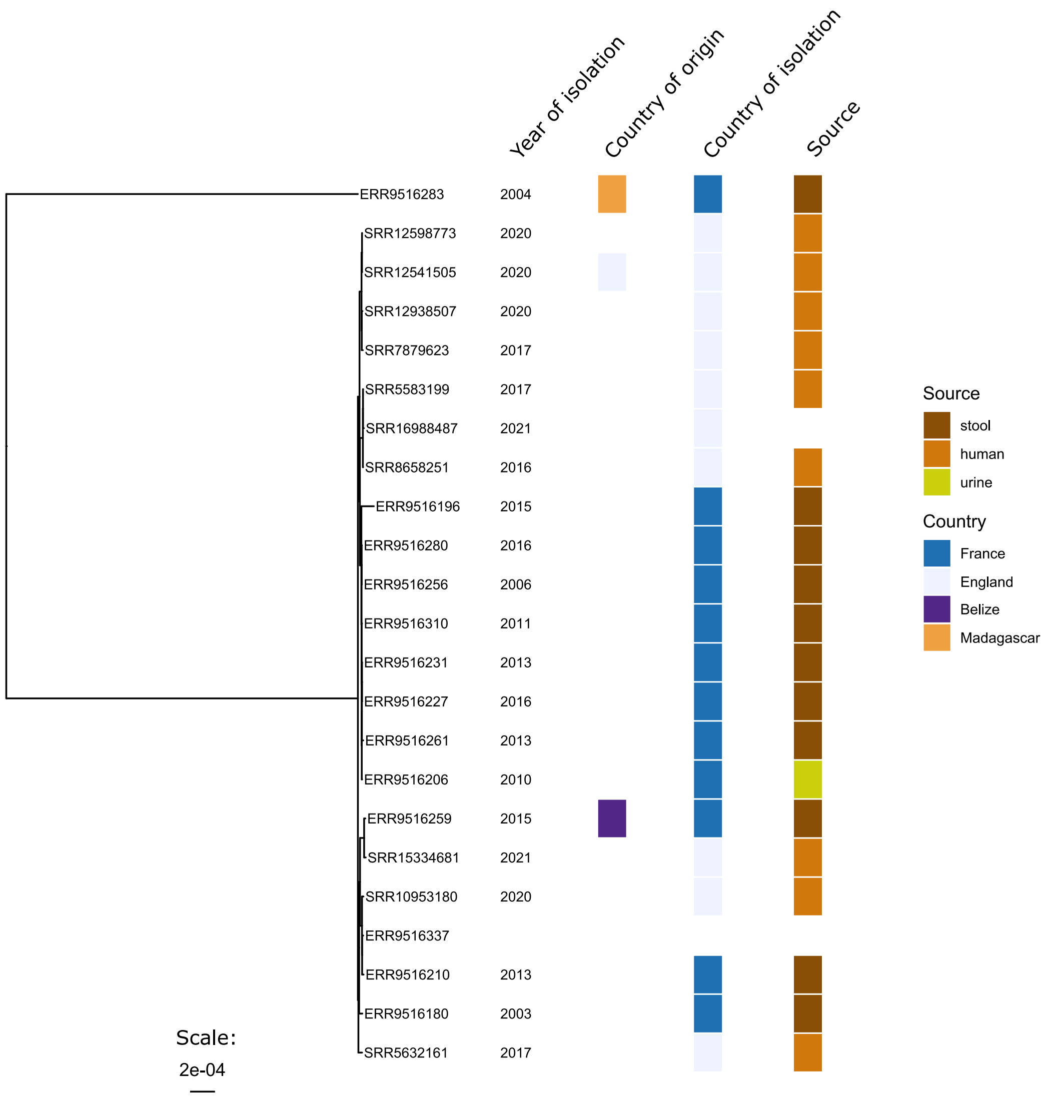

Supplementary Figure 1: S. Concord super-lineage B (HC2000_141)

Core gene maximum likelihood tree of *S*. Concord super-lineage B isolates. Leaves show the accession number. Complementary metadata is shown as text (year of isolation) or as a coloured track (country of origin, country of isolation and isolation source).

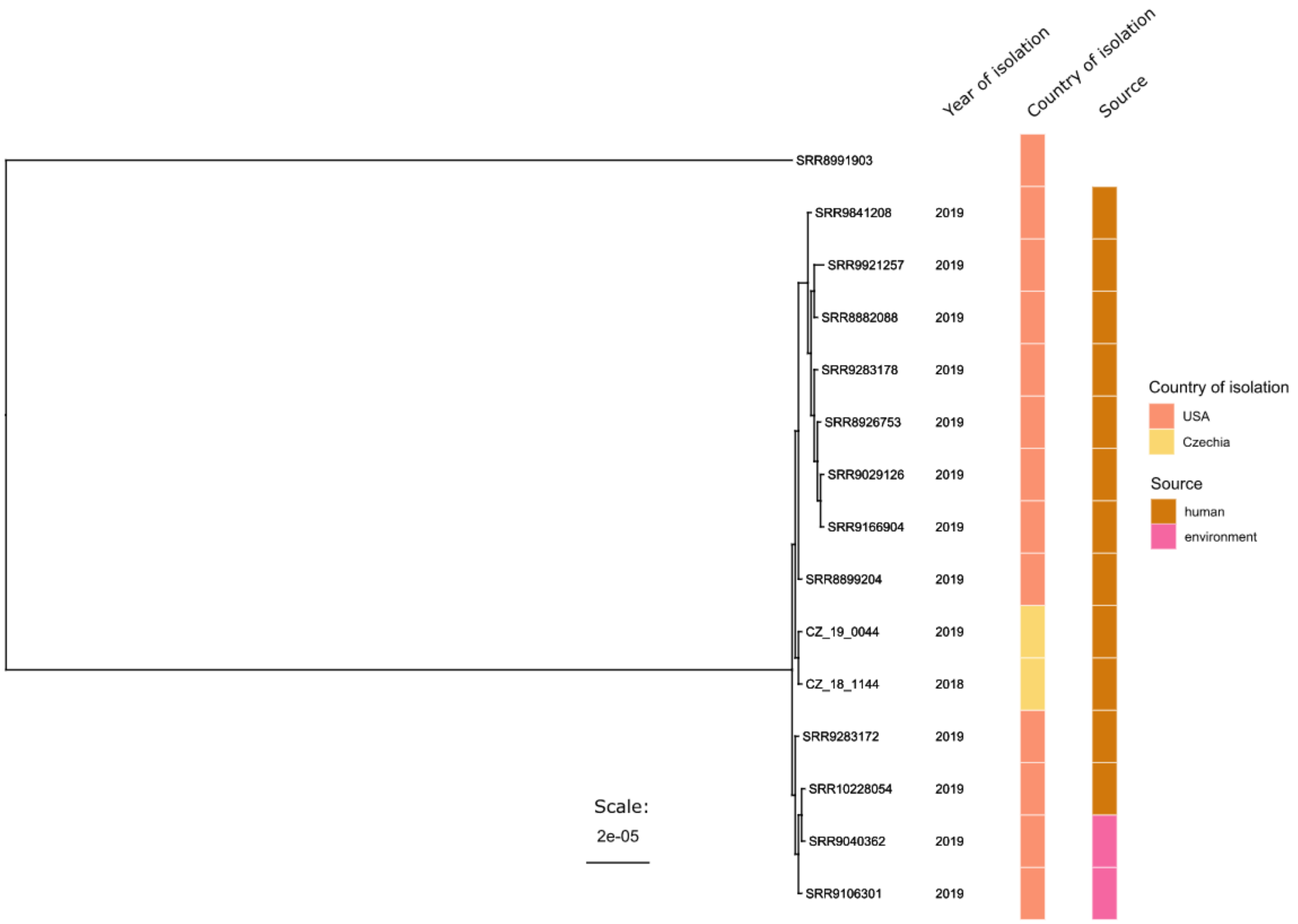

Supplementary Figure 2: *S*. Concord super-lineage C (HC2000_177997)

Core gene maximum likelihood tree of S. Concord super-lineage C. Leaves show the accession number or isolate ID. Complementary metadata is shown as text (year of isolation) or as a coloured track (country of isolation and isolation source).

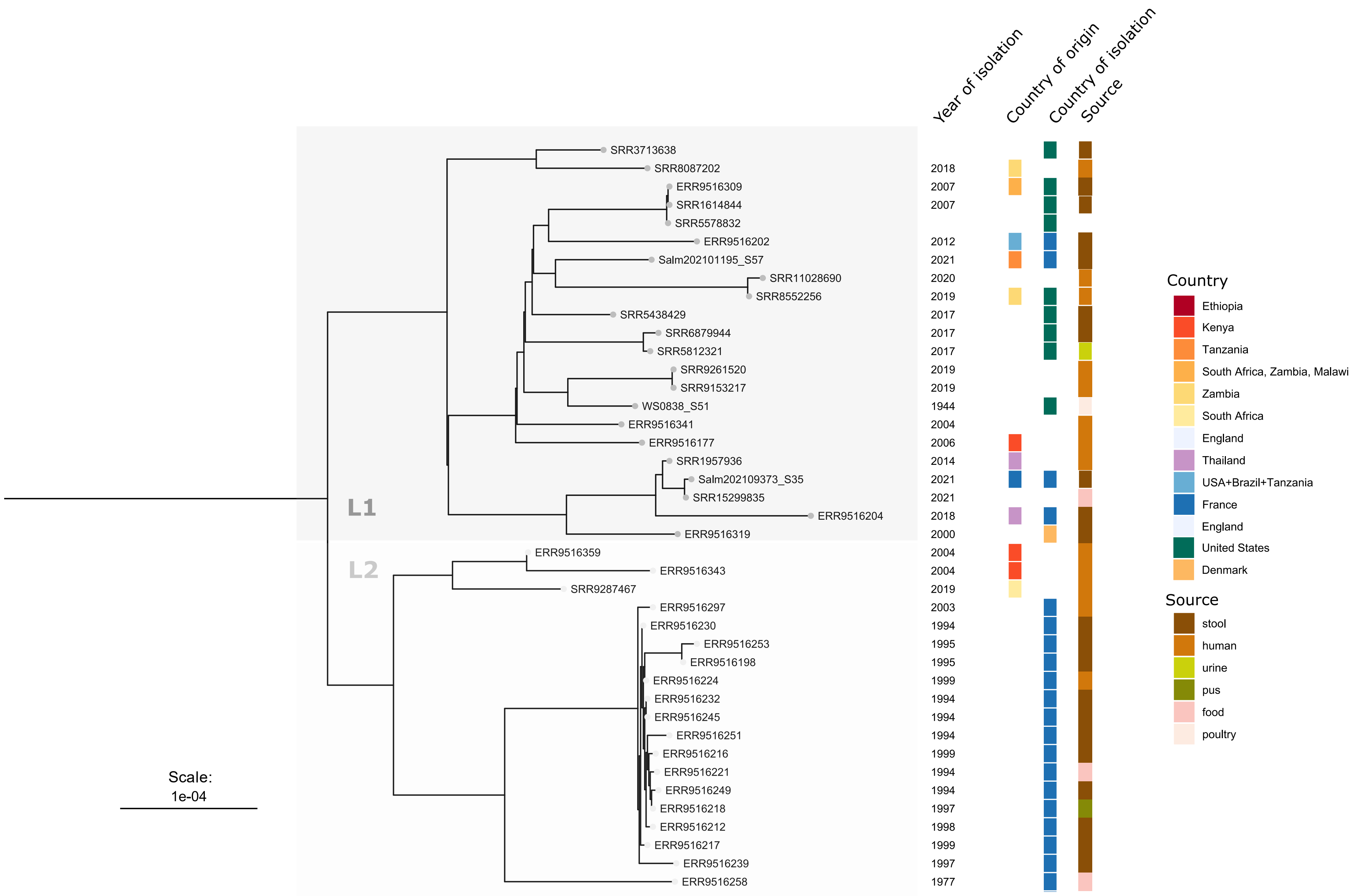
Year of isolation
Country of origin
Country of isolation
Source
SRR3713638
SRR8087202
ERR9516309
SRR1614844
SRR5578832
ERR9516202
Salm202101195_S57
SRR11028690
SRR8552256
SRR5438429
SRR6879944
SRR5812321
SRR9261520
SRR9153217
WS0838_S51
ERR9516341
ERR9516177
SRR1957936
Salm202109373_S35
SRR15299835
ERR9516204
ERR9516319
ERR9516359
ERR9516343
SRR9287467
ERR9516297
ERR9516230
ERR9516253
ERR9516198
ERR9516224
ERR9516232
ERR9516245
ERR9516251
ERR9516216
ERR9516221
ERR9516249
ERR9516218
ERR9516212
ERR9516217
ERR9516239
ERR9516258
L1
L2
2018
2007
2007
2012
2021
2020
2019
2017
2017
2017
2019
2019
1944
2004
2006
2014
2021
2021
2018
2000
2004
2004
2019
2003
1994
1995
1995
1999
1994
1994
1994
1999
1994
1994
1997
1998
1999
1997
1977
Scale:
1e-04
Country
Ethiopia
Kenya
Tanzania
South Africa, Zambia, Malawi
Zambia
South Africa
England
Thailand
USA+Brazil+Tanzania
France
England
United States
Denmark
Source
stool
human
urine
pus
food
poultry

Supplementary Figure 3: Detailed view of *S*. Concord super-lineage A lineages 1 (L1) and 2 (L2)

Leaves show the accession number of each isolate. Complementary metadata is shown as text (year of isolation) or as a coloured track (country of origin, country of isolation and isolation source).

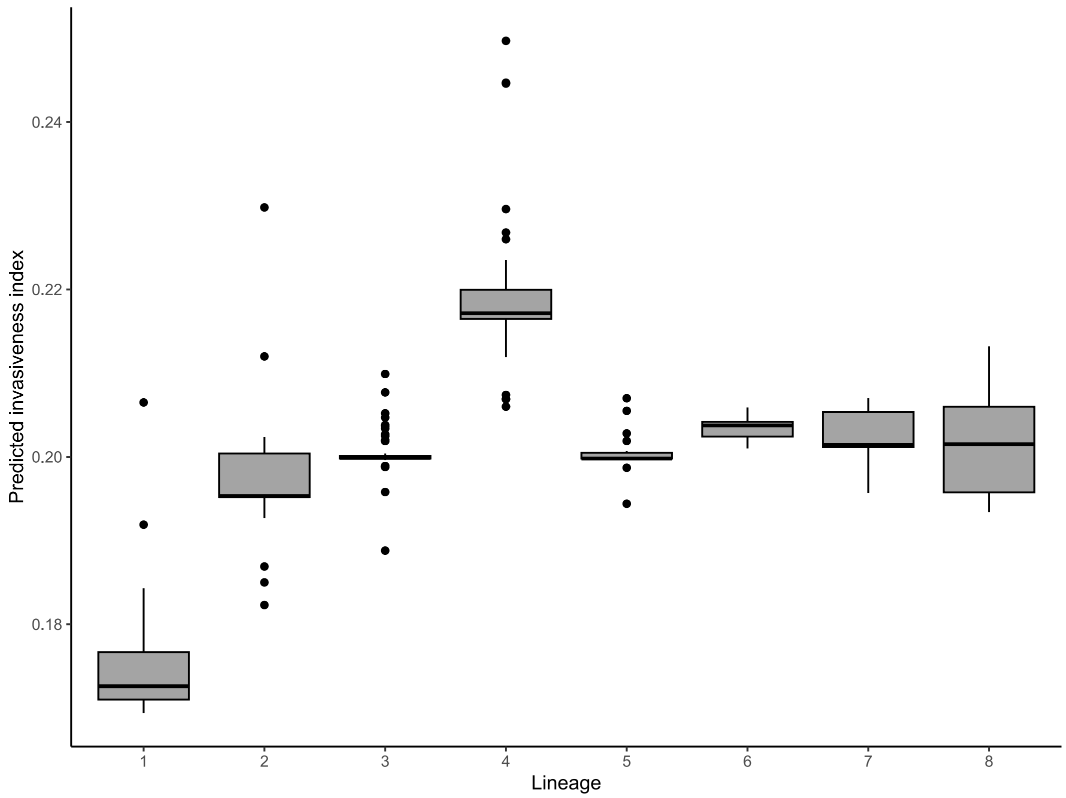

Supplementary Figure 4: Invasiveness index predicted for all *S*. Concord Super-lineage A isolates based on genomic features

Boxplot showing the distribution of the invasiveness index, as predicted per isolate by Wheeler et al.'s methodology (2018)[1]. Invasiveness index values (y-axis) were grouped per lineage (x-axis). Horizontal lines in each box represent the median value of the distribution, while the upper and lower quartiles are represented by the box limits. The whiskers extend to 1.5 times the interquartile range and individual data points beyond this range are considered outliers. The invasiveness index was predicted using a publicly available pipeline (https://github.com/Gardner-BinfLab/invasive_salmonella/tree/master/fastq_pipeline) that maps reads for each isolate onto a set of 196 genes previously linked to increased invasiveness, and generates a consensus protein sequence from the mapped reads for comparison to the same 196 genes using a functional variant calling metric[2]. The resulting features are then used as input for a pre-trained random forest classifier. The proportion of decision trees classifying an isolate as 'invasive' is referred to as the invasiveness index. Source data are provided as a Source Data file.

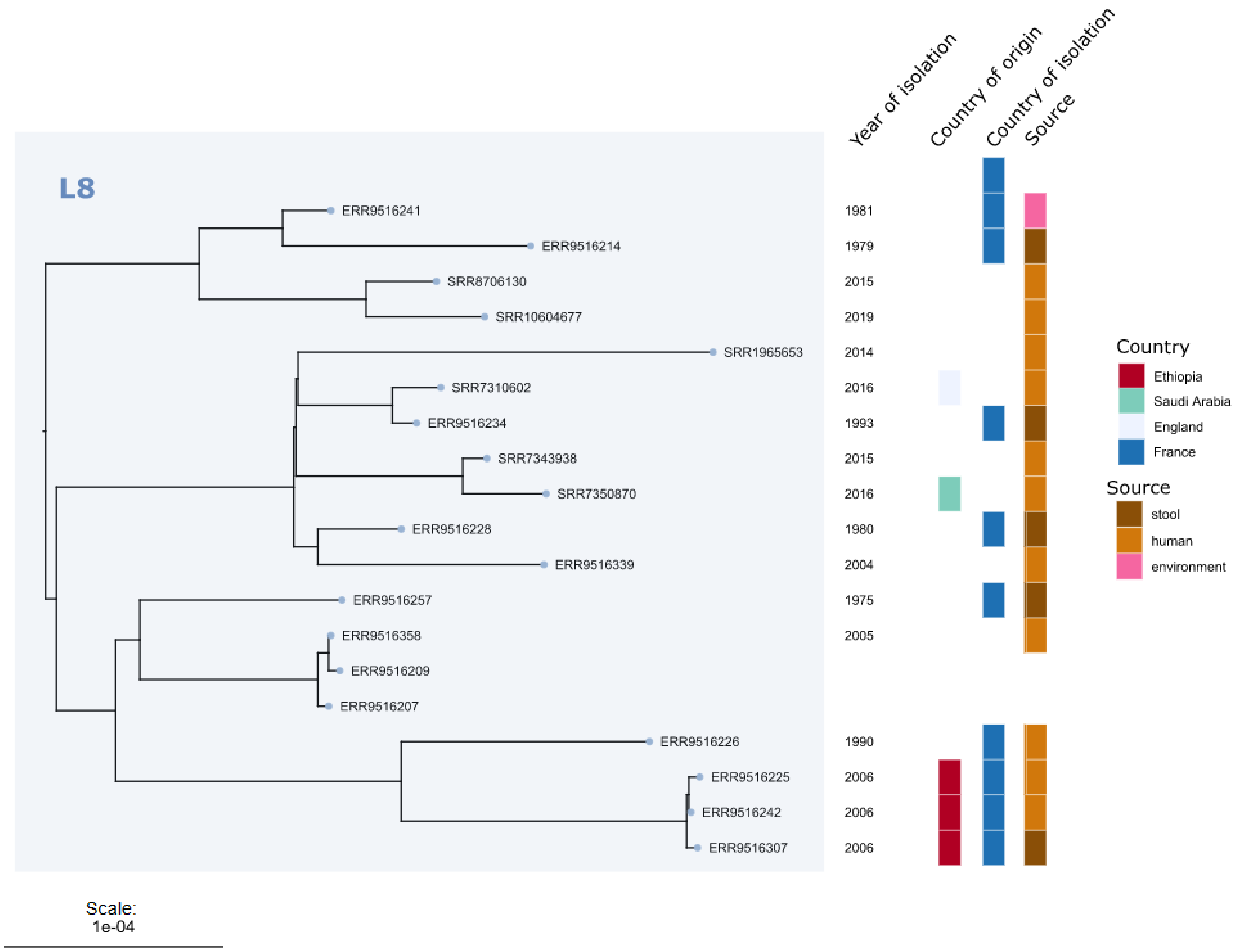
L8
ERR9516241
ERR9516214
SRR8706130
SRR10604677
SRR1965653
SRR7310602
ERR9516234
SRR7343938
SRR7350870
ERR9516228
ERR9516339
ERR9516257
ERR9516358
ERR9516209
ERR9516207
ERR9516226
ERR9516225
ERR9516242
ERR9516307
Year of isolation
Country of origin
Country of isolation
Source
1981
1979
2015
2019
2014
2016
1993
2015
2016
1980
2004
1975
2005
1990
2006
2006
2006
Country
Ethiopia
Saudi Arabia
England
France
Source
stool
human
environment
Scale:
1e-04

Supplementary Figure 5: Detail of super-lineage A lineage 8 (L8) showing the mixed occurrence of recent and historical isolates

Leaves show the accession number of each isolate. Complementary metadata is shown as text (year of isolation) or as a coloured track (country of origin, country of isolation and isolation source).

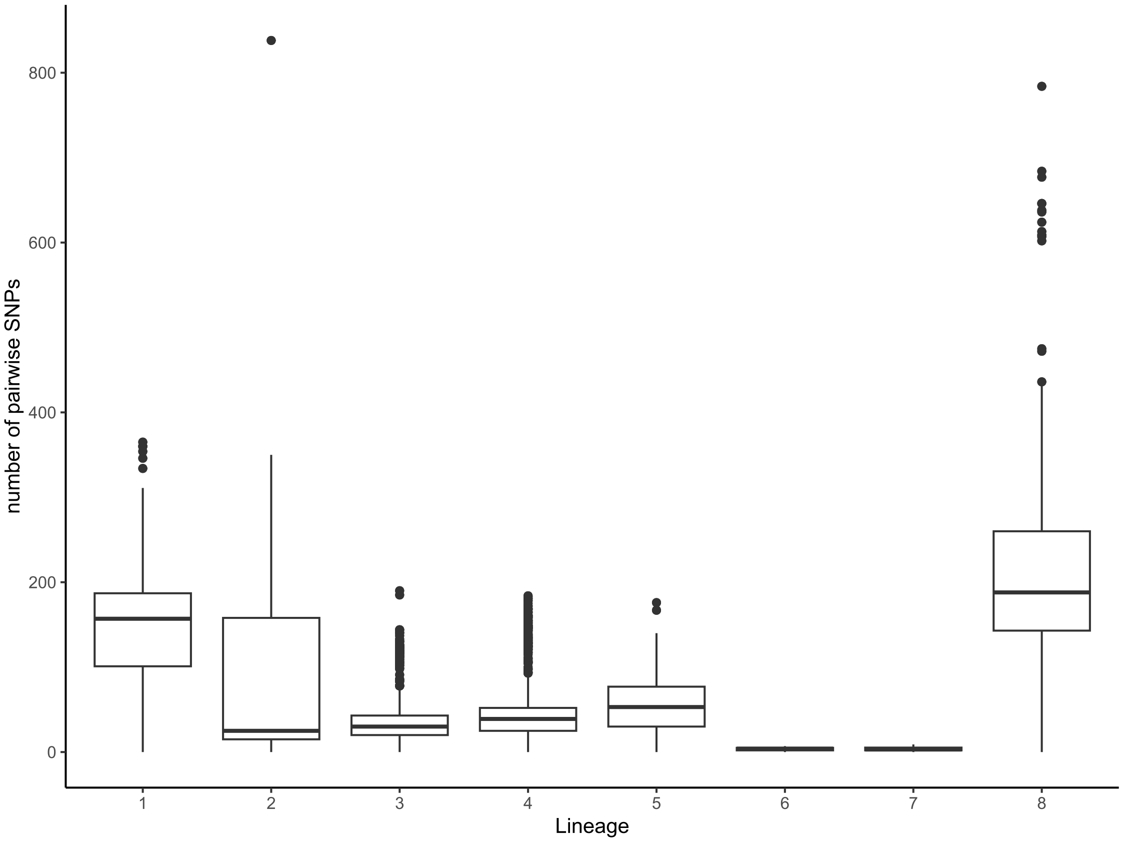

Supplementary Figure 6: Pairwise SNP differences within each *S*. Concord Super-lineage A lineage

Boxplot showing the distribution of pairwise SNP differences between all isolates within a given lineage. Pairwise SNP differences (y-axis) were calculated using snp-dists[3] v0.7.0 from the core SNP alignment used for phylogenetic inference prior to removing recombinant regions. Values were subsequently grouped per lineage (x-axis). Horizontal lines in each box represent the median value of the distribution, while the upper and lower quartiles are represented by the box limits. The whiskers extend to 1.5 times the interquartile range and individual data points beyond this range are considered outliers. Source data are provided as a Source Data file.

.

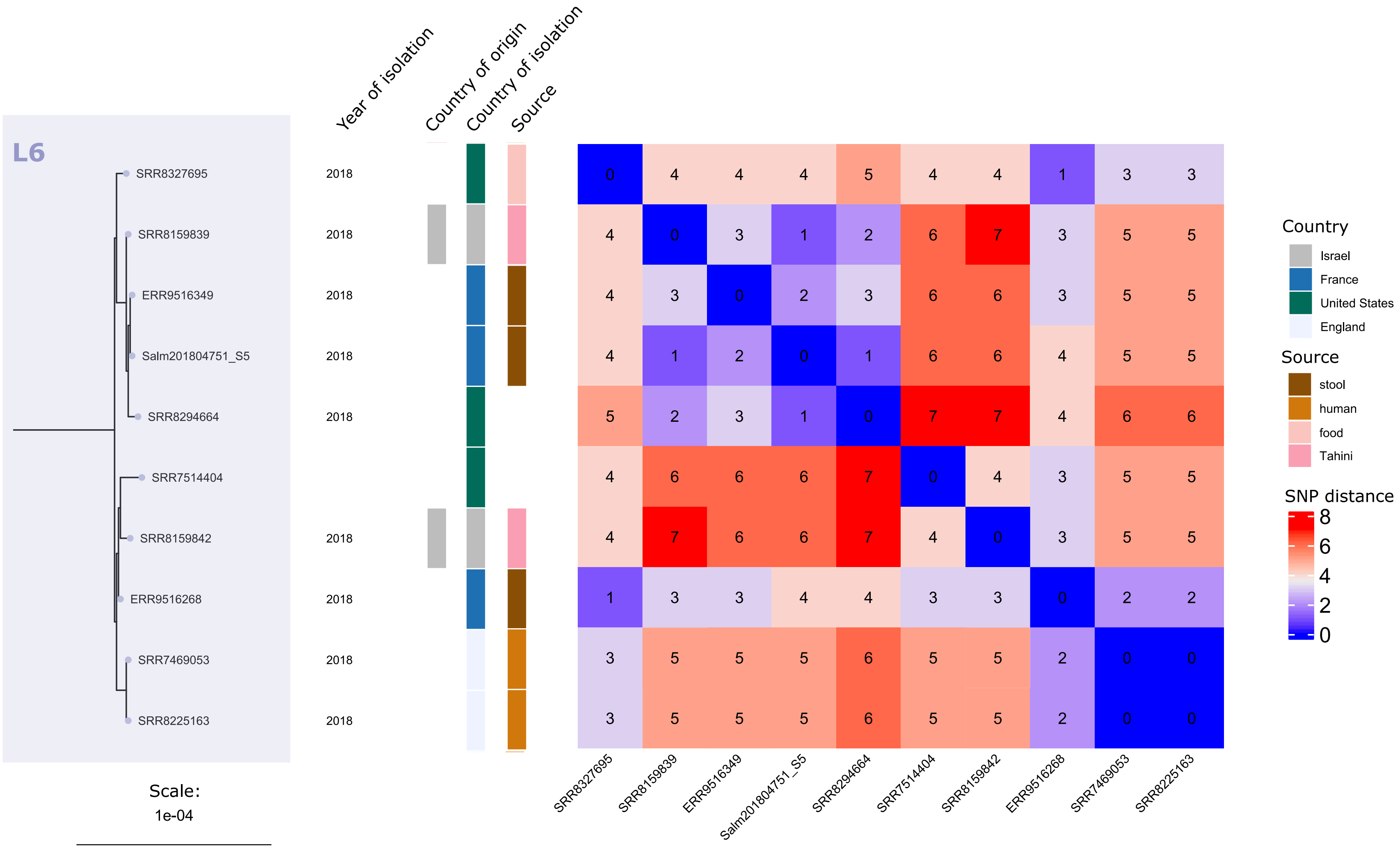
L6
Year of isolation
Country of origin
Country of isolation
Source
SRR8327695
SRR8159839
ERR9516349
Salm201804751_S5
SRR8294664
SRR7514404
SRR8159842
ERR9516268
SRR7469053
SRR8225163
2018
Scale:
1e-04
Country
Israel
France
United States
England
Source
stool
human
food
Tahini
SNP distance
8
6
4
2
0

Supplementary Figure 7: Detail of super-lineage A lineage 6 (L6) showing the close relatedness of isolates from Tahini isolated in Israel and clinical isolates

Leaves show the accession number of each isolate. Complementary metadata is shown as text (year of isolation) or as a coloured track (country of origin, country of isolation and isolation source). The heatmap on the right-hand side of the figure shows the SNP distance between all isolates

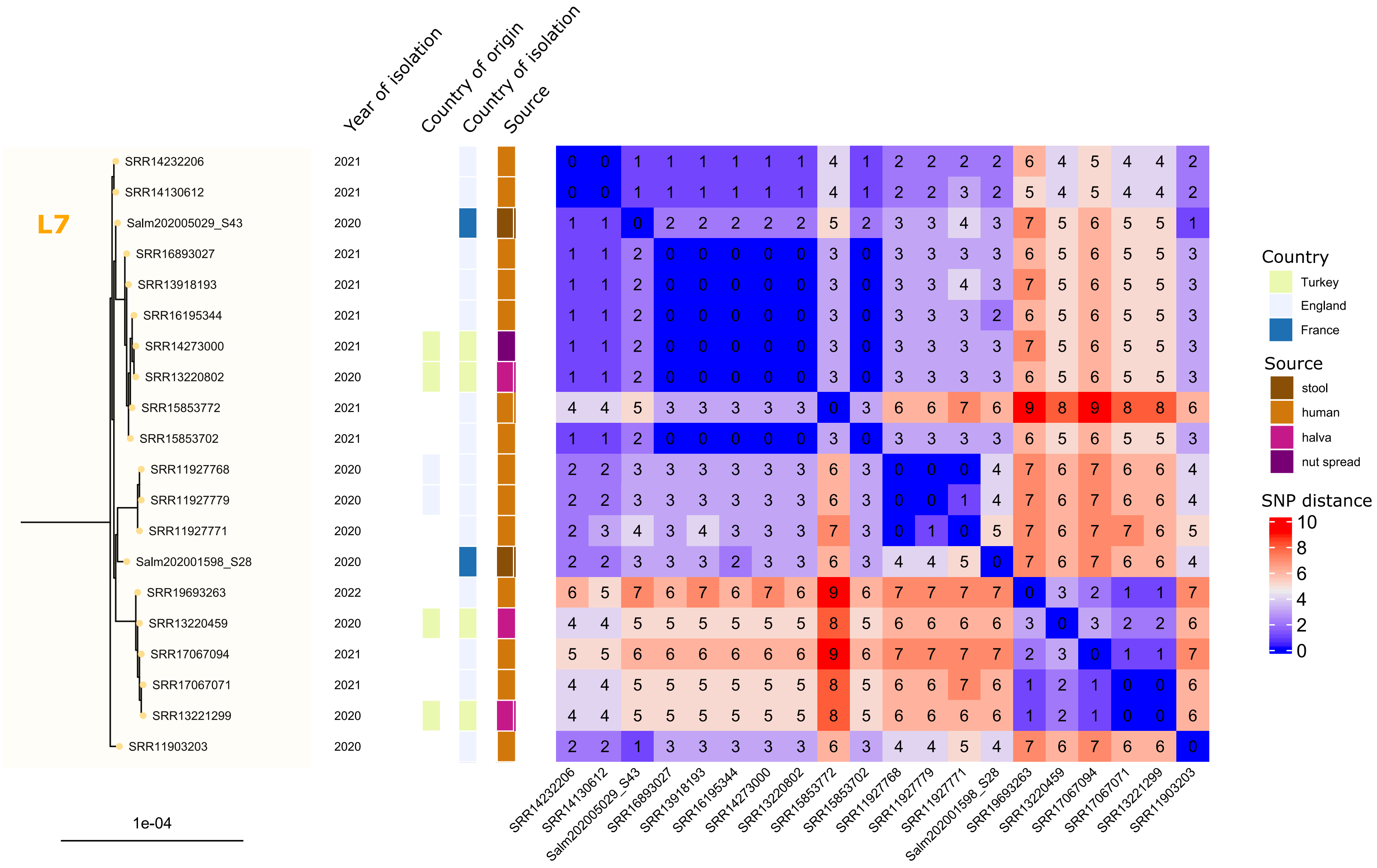
Year of isolation
Country of origin
Country of isolation
Source
L7
SRR14232206 2021
SRR14130612 2021
Salm202005029_S43 2020
SRR16893027 2021
SRR13918193 2021
SRR16195344 2021
SRR14273000 2021
SRR13220802 2020
SRR15853772 2021
SRR15853702 2021
SRR11927768 2020
SRR11927779 2020
SRR11927771 2020
Salm202001598_S28 2020
SRR19693263 2022
SRR13220459 2020
SRR17067094 2021
SRR17067071 2021
SRR13221299 2020
SRR11903203 2020
1e-04
0 0 1 1 1 1 1 1 4 1 2 2 2 2 6 4 5 4 4 2
0 0 1 1 1 1 1 1 4 1 2 2 3 2 5 4 5 4 4 2
1 1 0 2 2 2 2 2 5 2 3 3 4 3 7 5 6 5 5 1
1 1 2 0 0 0 0 0 3 0 3 3 3 3 6 5 6 5 5 3
1 1 2 0 0 0 0 0 3 0 3 3 4 3 7 5 6 5 5 3
1 1 2 0 0 0 0 0 3 0 3 3 3 2 6 5 6 5 5 3
1 1 2 0 0 0 0 0 3 0 3 3 3 3 7 5 6 5 5 3
1 1 2 0 0 0 0 0 3 0 3 3 3 3 6 5 6 5 5 3
4 4 5 3 3 3 3 3 0 3 6 6 7 6 9 8 9 8 8 6
1 1 2 0 0 0 0 0 3 0 3 3 3 3 6 5 6 5 5 3
2 2 3 3 3 3 3 3 6 3 0 0 0 4 7 6 7 6 6 4
2 2 3 3 3 3 3 3 6 3 0 0 1 4 7 6 7 6 6 4
2 3 4 3 4 3 3 3 7 3 0 1 0 5 7 6 7 7 6 5
2 2 3 3 3 2 3 3 6 3 4 4 5 0 7 6 7 6 6 4
6 5 7 6 7 6 7 6 9 6 7 7 7 7 0 3 2 1 1 7
4 4 5 5 5 5 5 5 8 5 6 6 6 6 3 0 3 2 2 6
5 5 6 6 6 6 6 6 9 6 7 7 7 7 2 3 0 1 1 7
4 4 5 5 5 5 5 5 8 5 6 6 7 6 1 2 1 0 0 6
4 4 5 5 5 5 5 5 8 5 6 6 6 6 1 2 1 0 0 6
2 2 1 3 3 3 3 3 6 3 4 4 5 4 7 6 7 6 6 0
SRR14232206
SRR14130612
Salm202005029_S43
SRR16893027
SRR13918193
SRR16195344
SRR14273000
SRR13220802
SRR15853772
SRR15853702
SRR11927768
SRR11927779
SRR11927771
Salm202001598_S28
SRR19693263
SRR13220459
SRR17067094
SRR17067071
SRR13221299
SRR11903203
Country
Turkey
England
France
Source
stool
human
halva
nut spread
SNP distance
10
8
6
4
2
0

Supplementary Figure 8: Close relatedness of clinical isolates and isolates from Halva food products from Turkey in super-lineage A lineage 7 (L7)

Leaves show the accession number of each isolate. Complementary metadata is shown as text (year of isolation) or as a coloured track (country of origin, country of isolation and isolation source). The heatmap on the right-hand side of the figure shows the SNP distance between all isolates.

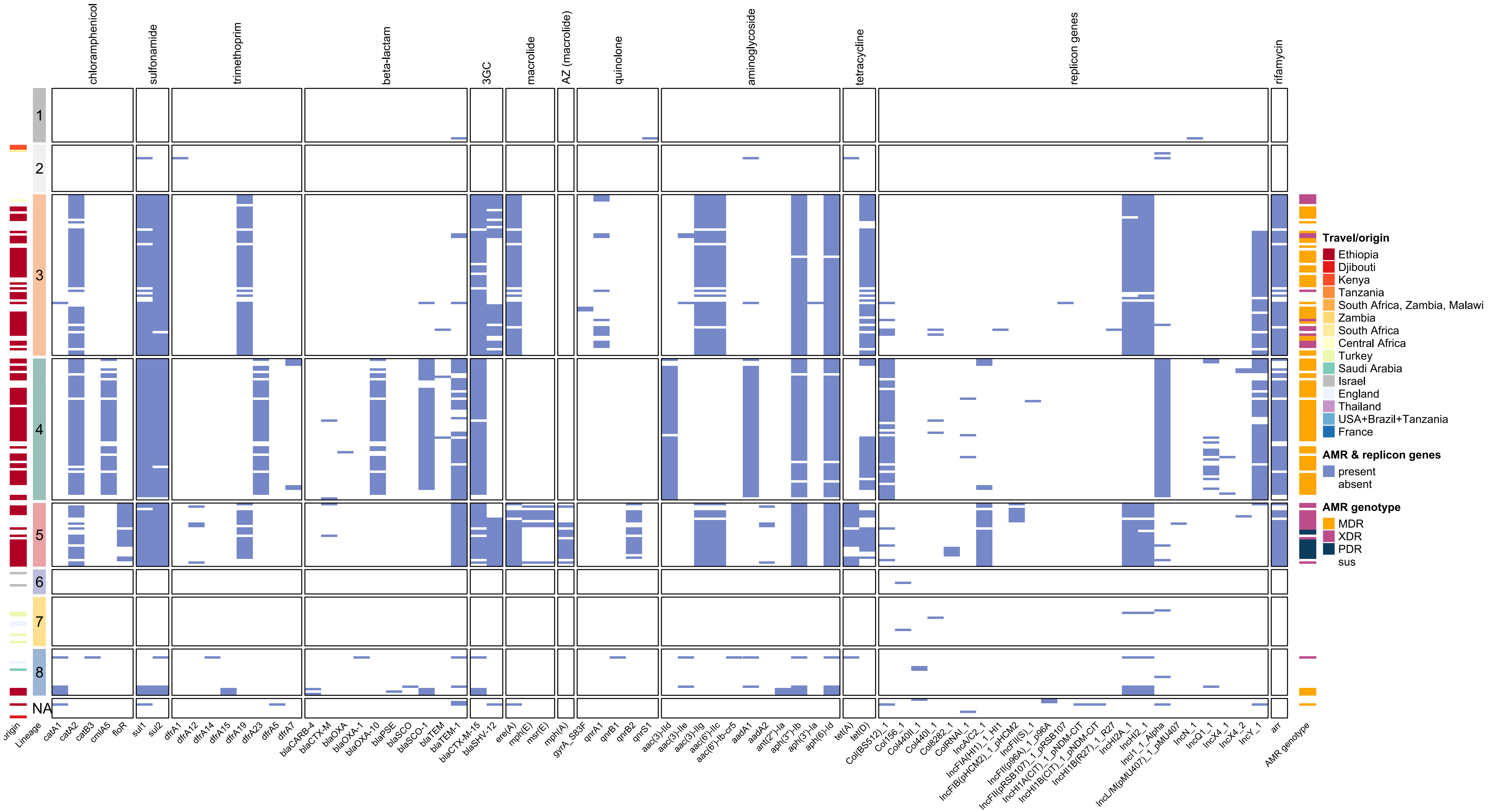

chloramphenicol
sulfonamide
trimethoprim
beta-lactam
3GC
macrolide
AZ (macrolide)
quinolone
aminoglycoside
tetracycline
replicon genes
rifamycin
1
2
3
4
5
6
7
8
NA
origin
Lineage
catA1
catA2
catB3
cmlA5
floR
sul1
sul2
dfrA1
dfrA12
dfrA14
dfrA15
dfrA19
dfrA23
dfrA5
dfrA7
blaCARB-4
blaCTX-M
blaOXA
blaOXA-1
blaOXA-10
blaPSE
blaSCO
blaSCO-1
blaTEM
blaTEM-1
blaCTX-M-15
blaSHV-12
ere(A)
mph(E)
msr(E)
mph(A)
gyrA_S83F
qnrA1
qnrB1
qnrB2
qnrS1
aac(3)-IId
aac(3)-IIe
aac(3)-IIg
aac(6')-IIc
aac(6')-Ib-cr5
aadA1
aadA2
ant(2'')-Ia
aph(3'')-Ib
aph(3')-Ia
aph(6)-Id
tet(A)
tet(D)
Col(BS512)_1
Col156_1
Col440II_1
Col440I_1
Col8282_1
ColRNAI_1
IncA/C2_1
IncFIA(HI1)_1_HI1
IncFIB(pHCM2)_1_pHCM2
IncFII(S)_1
IncFII(p96A)_1_p96A
IncFII(pRSB107)_1_pRSB107
IncHI1A(CIT)_1_pNDM-CIT
IncHI1B(CIT)_1_pNDM-CIT
IncHI1B(R27)_1_R27
IncHI2A_1
IncHI2_1
IncI1_1_Alpha
IncL/M(pMU407)_1_pMU407
IncN_1
IncQ1_1
IncX4_1
IncX4_2
IncY_1
arr
AMR genotype
Travel/origin
Ethiopia
Djibouti
Kenya
Tanzania
South Africa, Zambia, Malawi
Zambia
South Africa
Central Africa
Turkey
Saudi Arabia
Israel
England
Thailand
USA+Brazil+Tanzania
France
AMR & replicon genes
present
absent
AMR genotype
MDR
XDR
PDR
sus

Supplementary Figure 9: Complete set of AMR genes and plasmid replicon genes identified in *S*. Concord draft genomes, including AMR genes that do not contribute to the MDR, XDR and PDR phenotypes and less common replicon types

Presence (blue cells) or absence (white cells) of AMR genes identified in each *S*. Concord draft genomes using the AMRFinderPlus[4] tool is shown (only exact matches are shown and the default database was used). Lineages are shown at the left-hand side next to the heatmap, as well as the origin of the isolate. At the right-hand side, the genomic AMR combinations are shown (MDR, XDR and PDR). The ComplexHeatmap[5] package v2.8.0 for R was used to draw the heatmap. **Abbreviations**: AMR = antimicrobial resistance, MDR = multidrug resistance, ESBL = extended-spectrum beta-lactamase, XDR = extensive drug resistance, PDR = pandrug resistance, sus = susceptible, 3GC = third-generation cephalosporin, AZ = azithromycin. Raw data is included in **Supplementary Data 1**.

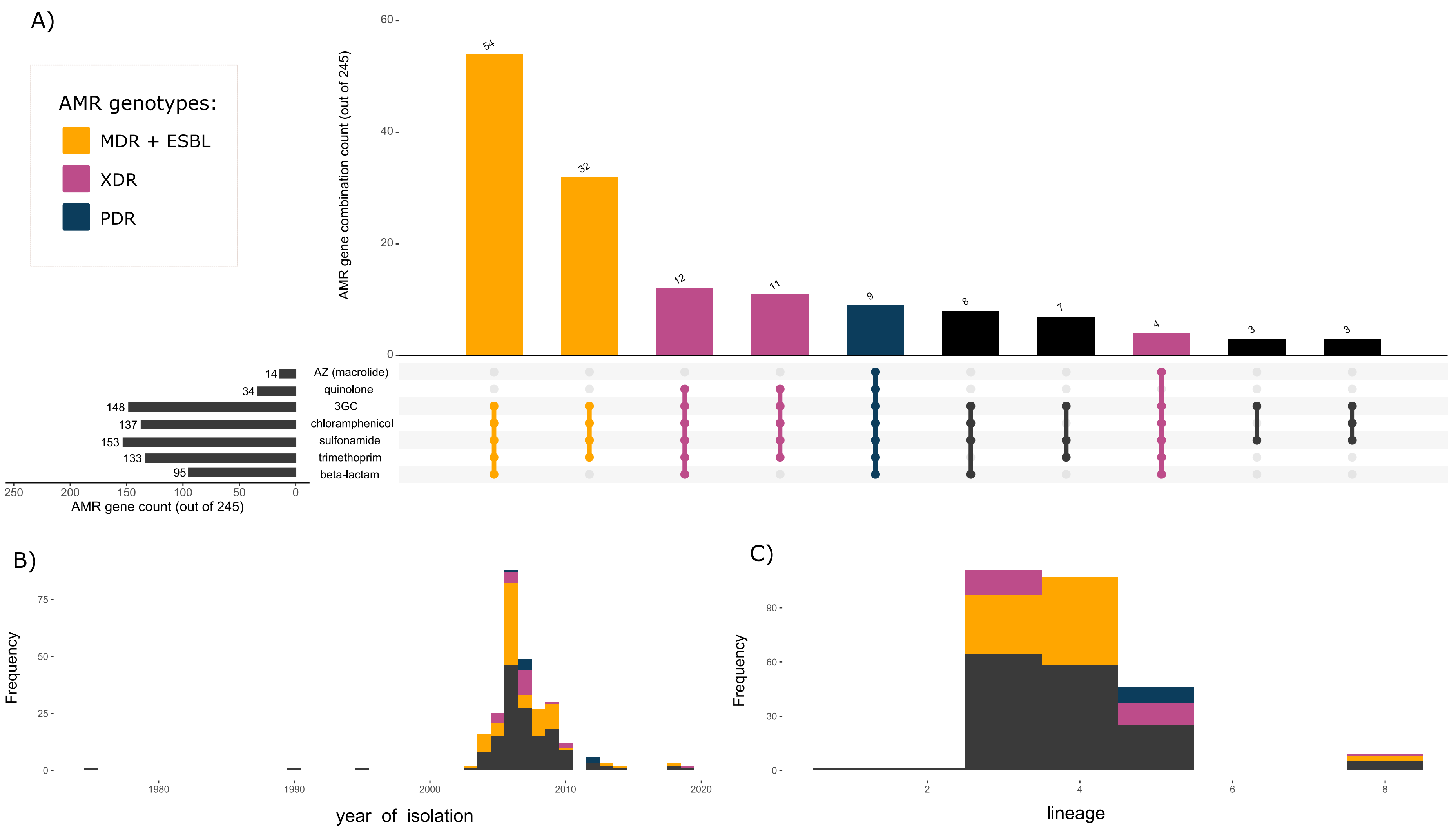
A)
AMR genotypes:
MDR + ESBL
XDR
PDR
AMR gene combination count (out of 245)
60
40
20
0
54
32
12
11
9
8
7
4
3
3
14
34
148
137
153
133
95
AZ (macrolide)
quinolone
3GC
chloramphenicol
sulfonamide
trimethoprim
beta-lactam
250
200
150
100
50
0
AMR gene count (out of 245)
B)
Frequency
75
50
25
0
1980
1990
2000
2010
2020
year of isolation
C)
Frequency
90
60
30
0
2
4
6
8
lineage

Supplementary Figure 10: Genomic AMR combinations in *S*. Concord super-lineage A

A) UpSet plot showing the different genomic AMR combinations in the entire *S*. Concord SLA dataset. The number of resistance genes linked to a certain antimicrobial category is shown in the left, horizontally oriented barplot. The vertically oriented bar plot shows the number of times specific AMR gene combinations were present encoding MDR, XDR and PDR.

B) Stacked barplot showing the frequency of the most common AMR gene combinations per year of isolation of the isolates. Combinations that are not classified as MDR + ESBL, XDR or PDR are shown in grey.

C) Stacked barplot showing the frequency of the most common AMR gene combinations per lineage. Combinations that are not classified as MDR + ESBL, XDR or PDR are shown in black.

The UpSetR[6] package v1.4.0 was used in R to construct the plots. **Abbreviations:** AMR = antimicrobial resistance, MDR = multidrug resistance, ESBL = extended-spectrum beta-lactamase, XDR = extensive drug resistance and PDR = pandrug resistance, AZ = azithromycin, 3GC = third-generation cephalosporin.

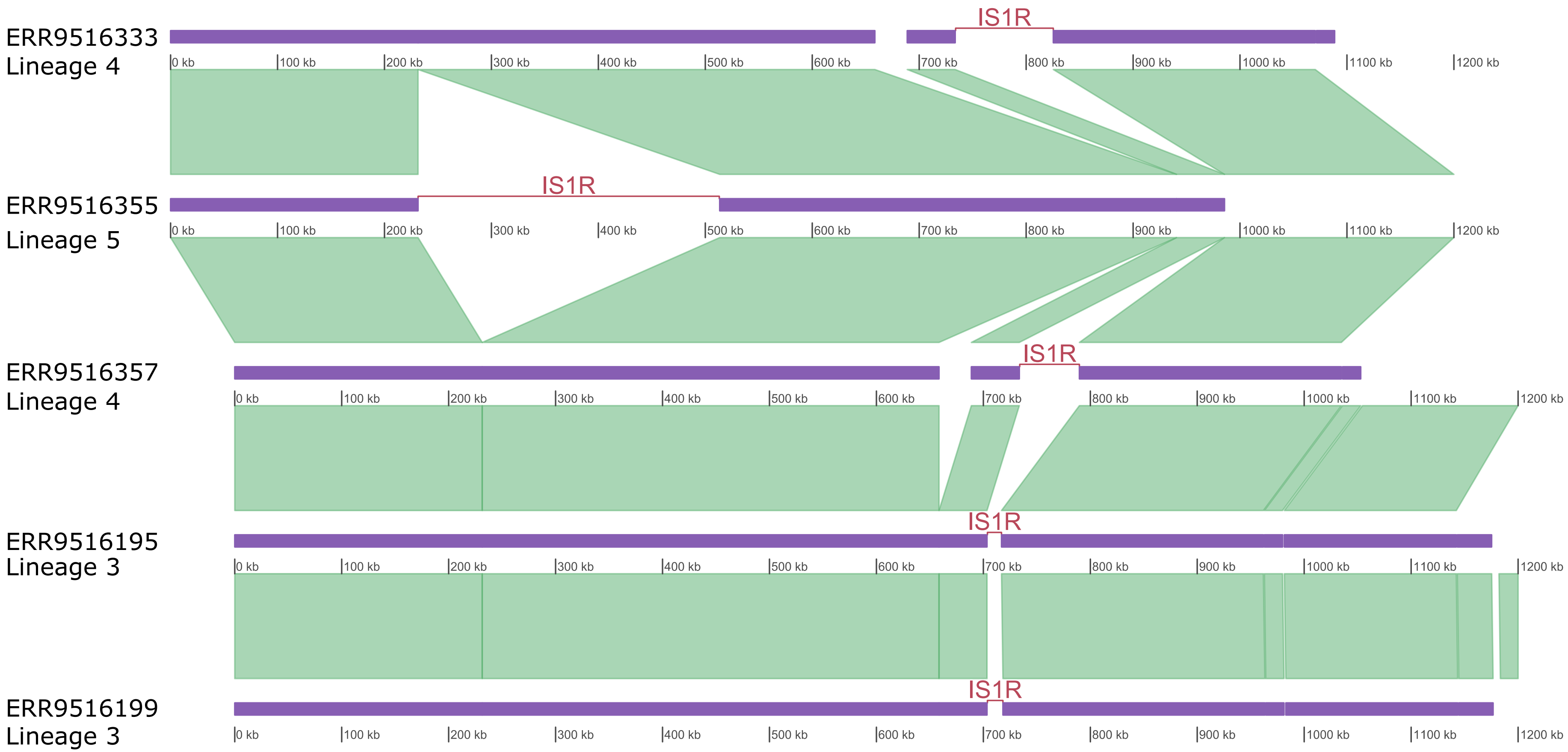
ERR9516333
Lineage 4
IS1R
0 kb
100 kb
200 kb
300 kb
400 kb
500 kb
600 kb
700 kb
800 kb
900 kb
1000 kb
1100 kb
1200 kb
ERR9516355
Lineage 5
IS1R
ERR9516357
Lineage 4
IS1R
ERR9516195
Lineage 3
IS1R
ERR9516199
Lineage 3
IS1R

Supplementary Figure 11: Chromosomal AMR cassettes flanked by IS*1R* were integrated at different positions in the long read sequenced *S.* Concord isolates and varied in length

Homologous regions conserved across the genomes were determined using Mauve[7] and visualised using the genoplotR[8] package v0.8.11 in R. Only the first 1200 kilobases of long read sequenced chromosomes containing antimicrobial resistance (AMR) genes integrated into the chromosome are shown. Purple blocks represent conserved sequences which are connected with green lines to visualize relative changes in position. The location of a chromosomally integrated AMR gene cassette flanked by IS*1R* transposase genes is highlighted in red. On the left-hand side the accession number of each isolate is shown in addition to the lineage were the isolate clusters.

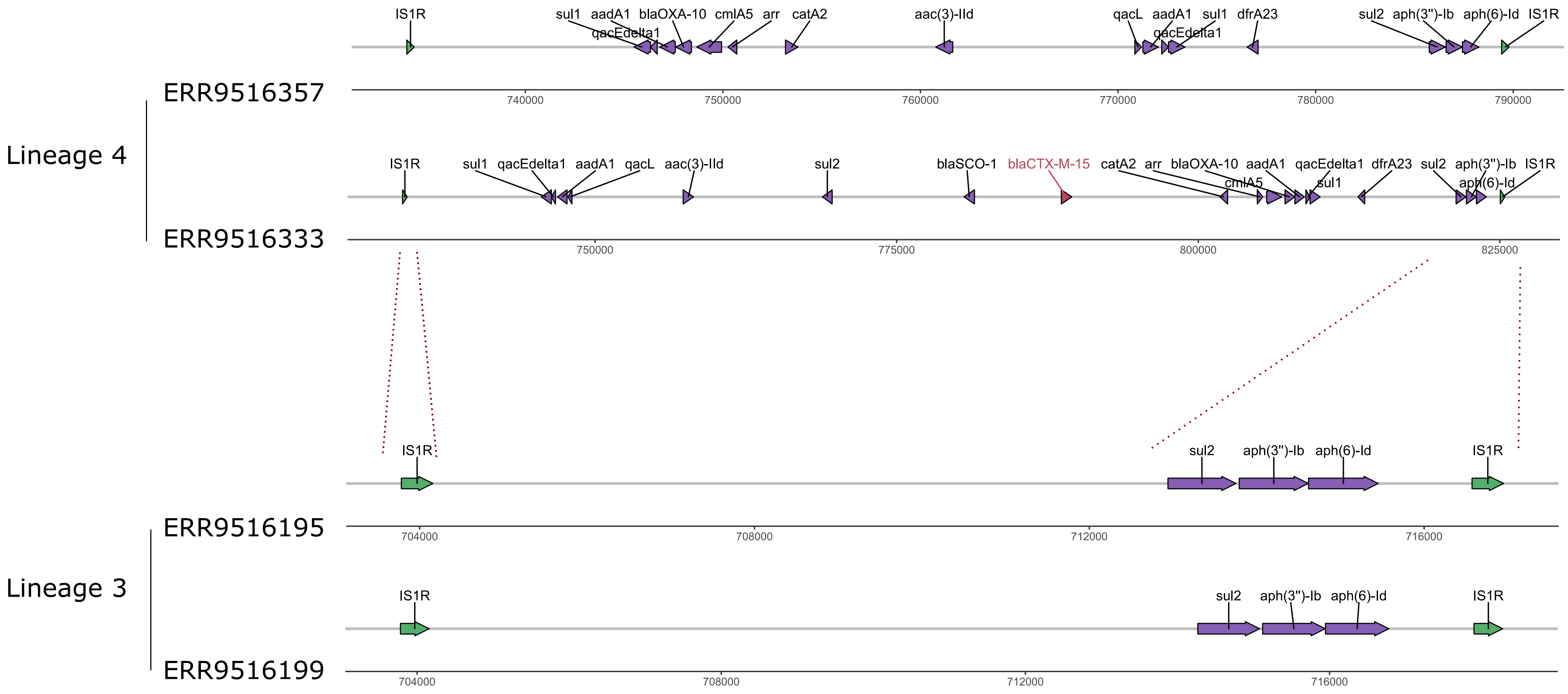
Lineage 4
ERR9516357
IS1R
sul1
aadA1
blaOXA-10
cmlA5
arr
catA2
qacEdelta1
aac(3)-IId
qacL
aadA1
sul1
dfrA23
qacEdelta1
sul2
aph(3'')-Ib
aph(6)-Id
IS1R
740000
750000
760000
770000
780000
790000
ERR9516333
IS1R
sul1
qacEdelta1
aadA1
qacL
aac(3)-IId
sul2
blaSCO-1
blaCTX-M-15
catA2
arr
blaOXA-10
cmlA5
aadA1
qacEdelta1
sul1
dfrA23
sul2
aph(3'')-Ib
aph(6)-Id
IS1R
750000
775000
800000
825000
Lineage 3
ERR9516195
IS1R
sul2
aph(3'')-Ib
aph(6)-Id
IS1R
704000
708000
712000
716000
ERR9516199
IS1R
sul2
aph(3'')-Ib
aph(6)-Id
IS1R
704000
708000
712000
716000

Supplementary Figure 12: Chromosomal AMR cassettes flanked by IS1R varied in composition in lineages 3 and 4

Detailed view of the AMR genes integrated into the *S*. Concord chromosome in reference genomes obtained from four L3 and L4 isolates. The ESBL gene bla$_{CTX\text{-}M\text{-}15}$ is highlighted in red. Other genes are linked to multidrug resistance and aminoglycoside resistance. Images were made using the R packages ggtree[9] and gggenes[10] and were combined and annotated in Inkscape v0.92.

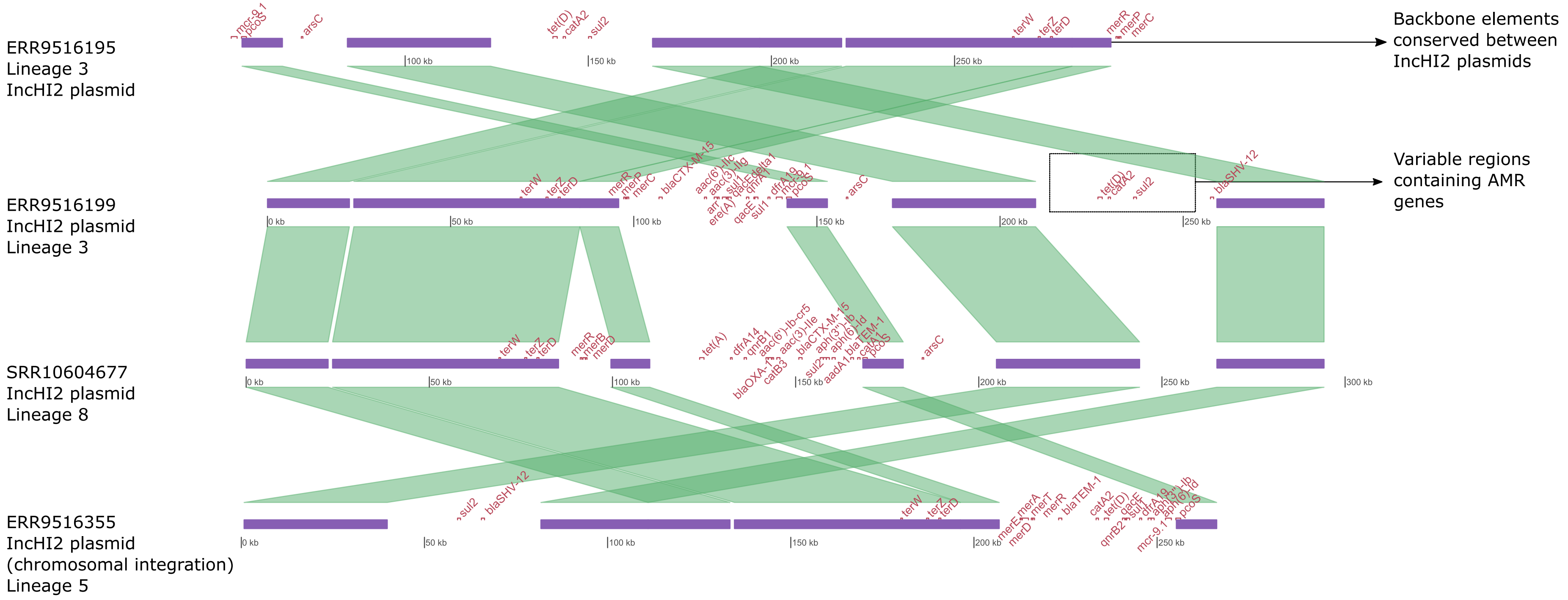

Supplementary Figure 13: IncHI2 plasmids and a chromosomally integrated IncHI2 plasmid shared common backbone elements and differed in regions encoding AMR

The alignment was made using mauve[7] and visualised using the genoplotR[8] package v0.8.11 in R. Text annotations in red are antimicrobial resistance genes and metal resistance genes identified using AMRFinderPlus[11] (default database).

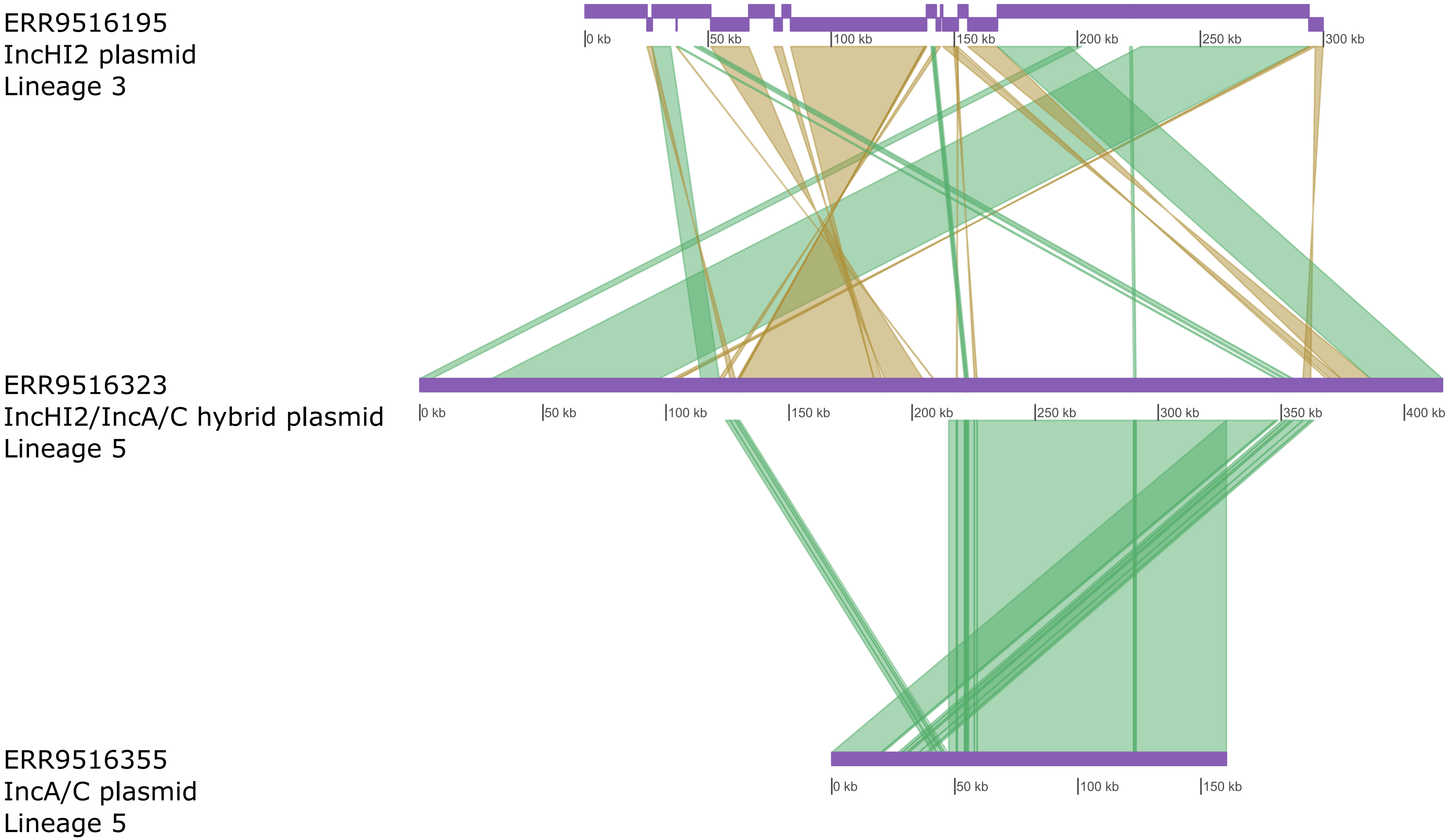
ERR9516195
IncHI2 plasmid
Lineage 3
0 kb
50 kb
100 kb
150 kb
200 kb
250 kb
300 kb
ERR9516323
IncHI2/IncA/C hybrid plasmid
Lineage 5
0 kb
50 kb
100 kb
150 kb
200 kb
250 kb
300 kb
350 kb
400 kb
ERR9516355
IncA/C plasmid
Lineage 5
0 kb
50 kb
100 kb
150 kb

Supplementary Figure 14: The XDR hybrid plasmid identified in isolate 32640_1_296 (middle track; part of Lineage 5) reference genome contained backbone elements derived from IncHI2 and IncA/C plasmids

The alignment was made using mauve[7] and visualised using the genoplotR[8] package v0.8.11 in R.

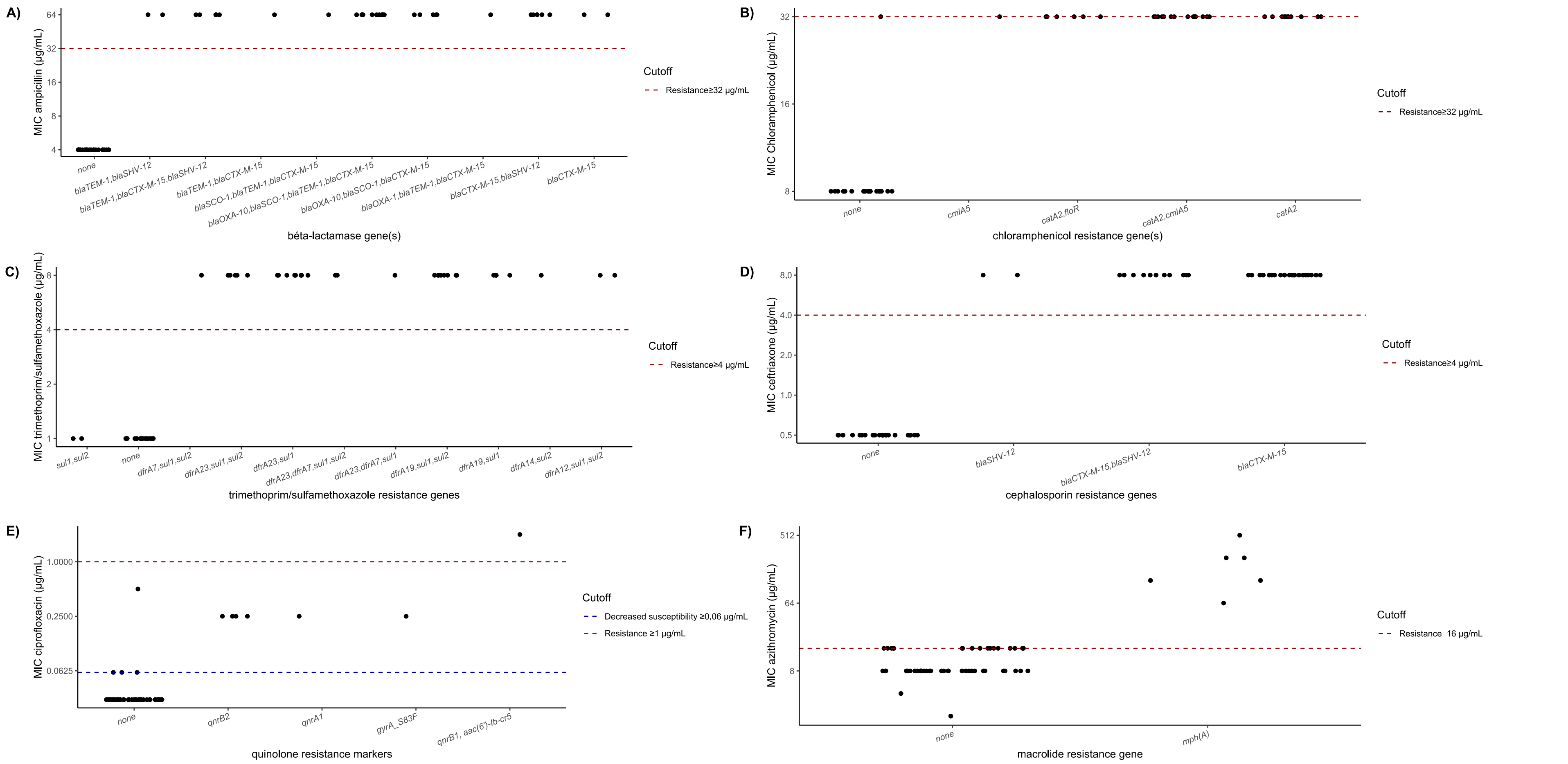
A)
MIC ampicillin (µg/mL)
64
32
16
8
4
none
blaTEM-1,blaSHV-12
blaTEM-1,blaCTX-M-15,blaSHV-12
blaTEM-1,blaCTX-M-15
blaSCO-1,blaTEM-1,blaCTX-M-15
blaOXA-10,blaSCO-1,blaTEM-1,blaCTX-M-15
blaOXA-10,blaSCO-1,blaCTX-M-15
blaOXA-1,blaTEM-1,blaCTX-M-15
blaCTX-M-15,blaSHV-12
blaCTX-M-15
béta-lactamase gene(s)
Cutoff
Resistance≥32 µg/mL
B)
MIC Chloramphenicol (µg/mL)
32
16
8
none
cmlA5
catA2,floR
catA2,cmlA5
catA2
chloramphenicol resistance gene(s)
Cutoff
Resistance≥32 µg/mL
C)
MIC trimethoprim/sulfamethoxazole (µg/mL)
8
4
2
1
sul1,sul2
none
dfrA7,sul1,sul2
dfrA23,sul1,sul2
dfrA23,sul1
dfrA23,dfrA7,sul1,sul2
dfrA23,dfrA7,sul1
dfrA19,sul1,sul2
dfrA19,sul1
dfrA14,sul2
dfrA12,sul1,sul2
trimethoprim/sulfamethoxazole resistance genes
Cutoff
Resistance≥4 µg/mL
D)
MIC ceftriaxone (µg/mL)
8.0
4.0
2.0
1.0
0.5
none
blaSHV-12
blaCTX-M-15,blaSHV-12
blaCTX-M-15
cephalosporin resistance genes
Cutoff
Resistance≥4 µg/mL
E)
MIC ciprofloxacin (µg/mL)
1.0000
0.2500
0.0625
none
qnrB2
qnrA1
gyrA_S83F
qnrB1, aac(6')-Ib-cr5
quinolone resistance markers
Cutoff
Decreased susceptibility ≥0.06 µg/mL
Resistance ≥1 µg/mL
F)
MIC azithromycin (µg/mL)
512
64
8
none
mph(A)
macrolide resistance gene
Cutoff
Resistance 16 µg/mL

Supplementary Figure 15: Association of AMR genotype and phenotype for first- and second-line antimicrobials recommended to treat invasive *Salmonella* infections

Genotype-phenotype association for ampicillin (A), chloramphenicol (B), trimethoprim-sulfamethoxazole (C), ceftriaxone (D), ciprofloxacin (E) and azithromycin (D). Dots indicate the minimum inhibitory concentration (MIC) (y-axis) for each of 56 isolates harbouring a certain combination of genomic resistance markers (shown on the x- axis). The dashed line in red indicates the CLSI M100-ED31 breakpoint for clinical resistance. For azithromycin the epidemiological cutoff was indicated by the dashed line in red. For ciprofloxacin, the dashed line in **blue** indicates the decreased ciprofloxacin susceptibility cutoff. Tested concentration ranges and measurements are listed in **Supplementary Data 2**.

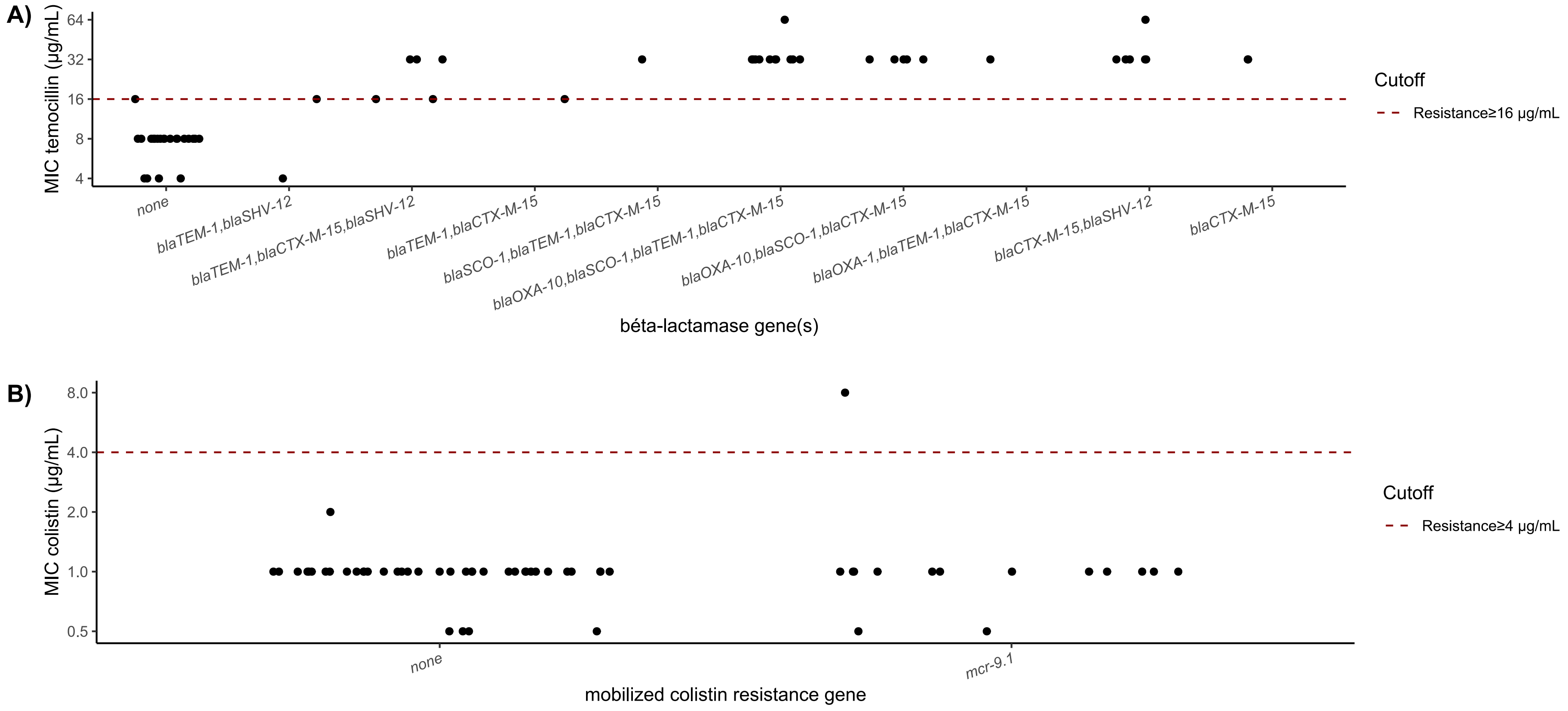
A)
MIC temocillin (µg/mL)
64
32
16
8
4
none
blaTEM-1,blaSHV-12
blaTEM-1,blaCTX-M-15,blaSHV-12
blaTEM-1,blaCTX-M-15
blaSCO-1,blaTEM-1,blaCTX-M-15
blaOXA-10,blaSCO-1,blaTEM-1,blaCTX-M-15
blaOXA-10,blaSCO-1,blaCTX-M-15
blaOXA-1,blaTEM-1,blaCTX-M-15
blaCTX-M-15,blaSHV-12
blaCTX-M-15
béta-lactamase gene(s)
Cutoff
Resistance≥16 µg/mL
B)
MIC colistin (µg/mL)
8.0
4.0
2.0
1.0
0.5
none
mcr-9.1
mobilized colistin resistance gene
Cutoff
Resistance≥4 µg/mL

Supplementary Figure 16: Association of AMR genotype and phenotype for alternative antimicrobials

Genotype-phenotype association for temocillin (A) and colistin (B). Dots indicate the minimum inhibitory concentration (MIC) (y-axis) for each of 56 isolates harbouring a certain combination of genomic resistance markers (shown on the x- axis). The dashed line in red indicates the EFSA epidemiological cutoff for temocillin and the CLSI M100-ED31 breakpoint for clinical resistance for colistin. Tested concentration ranges and measurements are listed in **Supplementary Data 2.**

Supplementary Tables

Supplementary Table 1: Presence-absence variations in genes linked to increased invasive potential in L4 isolates. The number of genes that were missing (*i.e.* no significant BLAST hit) from at least one isolate from lineage 4 (L4) were reported. The full dataset was generated by comparing all annotated genes in the *S*. Concord draft assemblies to the set of 196 genes predictive of an invasive phenotype utilized in the machine learning approach by Wheeler et al. (2018), using BLAST[12,13] v2.13.0+. Per query sequence, the hit with the highest bit score was retained, and we jointly assessed the query coverage and percentage identity by calculating their product and multiplying by 100. Source data are provided as a Source Data file.

ID	Gene	Number of L4 isolates missing the gene	Full gene name
**STM1154**	*mdtG*	12	Multidrug resistance protein MdtG
**STM2245**	-	12	Putative outer membrane protein
**STM1349**	*pps*	8	Phosphoenolpyruvate synthase [EC=2.7.9.2]
**STM1338**	*pheT*	5	Phenylalanine--tRNA ligase beta subunit [EC=6.1.1.20]
**STM2066**	*sopA*	5	E3 ubiquitin-protein ligase SopA [EC=6.3.2.-]
**STM3152**	*mcpB*	5	Putative methyl-accepting chemotaxis protein
**SG1259**	-	3	Putative uncharacterized protein
**STM2808**	*nrdF*	3	Ribonucleoside-diphosphate reductase 2 subunit beta [EC=1.17.4.1]
**SEN3623**	*yidE*	1	Putative transport protein YidE
**STM0631**	*ybeM*	1	Putative hydrolase
**STM0994**	*mukB*	1	Chromosome partition protein MukB
**STM1746.S**	*oppA*	1	Periplasmic oligopeptide-binding protein
**STM1914**	*flhB*	1	Flagellar biosynthetic protein FlhB
**STM2036**	*pocR*	1	Regulatory protein PocR
**STM2043**	*pduG*	1	Propanediol utilization diol dehydratase reactivation protein
**STM2053**	*pduS*	1	Propanediol utilization protein
**STM2057**	*pduW*	1	Probable propionate kinase [EC=2.7.2.15]
**STM2065**	*phsA*	1	Thiosulfate reductase [EC=1.-.-.-]
**STM2481**	*acrD*	1	RND family aminoglycoside/multidrug efflux pump
**STM2815**	*emrB*	1	Putative MFS superfamily multidrug transport protein
**STM3125**	-	1	Putative cytoplasmic protein
**STM3789**	*uhpB*	1	Sensor protein UhpB [EC=2.7.13.3]
**STM4190**	*pepE*	1	Peptidase E [EC=3.4.13.21]

Supplementary Table 2: Comparison of genotypic and phenotypic resistance or susceptibility for antimicrobials that can be used to treat invasive *Salmonella* infections. Phenotypic resistance was determined using Sensititre microbroth dilution. An isolate was classified genotypically resistant if it harboured one genotypic resistance marker or more. (*) For ciprofloxacin, decreased ciprofloxacin susceptibility was counted as "resistant". Genotypic data is listed in **Supplementary Data 1**. Phenotypic data is listed in **Supplementary Data 2**.

Antimicrobial	n° isolates tested	Phenotype susceptible		Phenotype resistant*		Concordance (%)
		Genotype resistant	Genotype susceptible	Genotype resistant	Genotype susceptible	
**Ampicillin**	56	0	22	34	0	100,00
**Trimethoprim/Sulfamethoxazole**	56	0	24	32	0	100,00
**Chloramphenicol**	56	0	22	33	1	98,21
**Ceftriaxone**	56	0	22	34	0	100,00
**Ciprofloxacin**	56	0	48	7	1	98,21
**Azithromycin**	56	0	50	6	0	100,00

Supplementary Table 3: Assembly summary of long read sequenced genomes.

The table is continued on the following page. Abbreviations: ONT = Oxford Nanopore Technologies, PacBio = Pacific Biosciences

Sequencing technology	Sequencing id	Strain	Contig type	Contig size (bp)	Depth of coverage	AMR genes
ONT	32640_1_296	0508H45184	Chromosome	4700258	157.15	no
			pSC_0508H45184 IncHI2A + IncA/C2	415479	403.47	yes
ONT	32640_1_316	1035531	Chromosome	4899417	631.26	yes
			pSC_1035531_1	100933	584.52	yes
			pSC_1035531_2 IncI1_Alpha	85904	1570.08	no
ONT	32640_1_328	H04340470	Chromosome	4751619	470.26	no
			pSC_H04340470	4028	1181.62	no
ONT	32640_1_336	H044240362	Chromosome	4660598	805.32	no
ONT	32640_1_364	70366	Chromosome	5009183	471.68	yes
			pSC_70366_1 IncA/C2	160357	483.10	yes
			pSC_70366_2	92366	276.92	no
ONT	32640_1_372	254833	Chromosome	4864180	45.82	yes
			pSC_254833_1	13181	349.20	yes
			pSC_254833_2 IncA/C2	71024	138.74	yes
			pSC_254833_3	100933	43.96	yes
			pSC_254833_4 IncI1_Alpha	85904	113.74	no
ONT	32640_1_39	64206	Chromosome	4800102	270.25	yes
			pSC_64206_1 IncHI2_1	372354	464.49	yes
			pSC_64206_2	106569	306.49	no
			pSC_64206_3	47794	438.69	no
			pSC_64206_4	5350	2375.96	no

Supplementary Table 3 continued

Sequencing technology	Sequencing id	Strain	Contig type	Contig size (bp)	Depth of coverage	AMR genes
ONT	32640_1_47	95907	Chromosome	4818922	186.13	yes
			pSC_95907_1 IncHI2A_1	288445	298.45	yes
			pSC_95907_2	106569	192.63	no
			pSC_95907_3	93718	136.40	no
			pSC_95907_4	47794	385.39	no
			pSC_95907_5	5350	735.74	no
ONT	SRS4345282	679052	Chromosome	4657242	296.78	no
ONT	SRS5777896	850890	Chromosome	4670214	138.10	no
			pSC_850890 IncHI2A	294257	171.69	yes
PacBio	Mapping reference ITM_8091960	1309	Chromosome	4811936	108.08	yes
			pSC_1309_1 IncHI2A	276676	191.66	yes
			pSC_1309_2	106569	193.88	no
			pSC_1309_3	93719	197.52	no
			uncircularised_contig	78477	163.25	no
			str1309_pSC_1309_4	5354	252.24	no

References

1. Wheeler, N. E., Gardner, P. P. & Barquist, L. Machine learning identifies signatures of host adaptation in the bacterial pathogen Salmonella enterica. *PLoS Genet.* **14**, e1007333 (2018).
2. Wheeler, N. E., Barquist, L., Kingsley, R. A. & Gardner, P. P. A profile-based method for identifying functional divergence of orthologous genes in bacterial genomes. *Bioinformatics* **32**, 3566–3574 (2016).
3. Seemann, T. *snp-dists: Pairwise SNP distance matrix from a FASTA sequence alignment*. (Github).
4. Feldgarden, M. *et al.* Validating the AMRFinder tool and resistance gene database by using antimicrobial resistance genotype-phenotype correlations in a collection of isolates. *Antimicrob. Agents Chemother.* **63**, (2019).
5. Gu, Z., Eils, R. & Schlesner, M. Complex heatmaps reveal patterns and correlations in multidimensional genomic data. *Bioinformatics* **32**, 2847–2849 (2016).
6. Conway, J. R., Lex, A. & Gehlenborg, N. UpSetR: an R package for the visualization of intersecting sets and their properties. *Bioinformatics* **33**, 2938–2940 (2017).
7. Darling, A. C. E., Mau, B., Blattner, F. R. & Perna, N. T. Mauve: multiple alignment of conserved genomic sequence with rearrangements. *Genome Res.* **14**, 1394–1403 (2004).
8. Guy, L., Roat Kultima, J. & Andersson, S. G. E. genoPlotR: comparative gene and genome visualization in R. *Bioinformatics* vol. **26** 2334–2335 (2010).
9. Yu, G., Smith, D. K., Zhu, H., Guan, Y. & Lam, T. T. ggtree : an r package for visualization and annotation of phylogenetic trees with their covariates and other associated data. *Methods in Ecology and Evolution* vol. **8** 28–36 (2017).
10. Wilkins, D. gggenes. https://github.com/wilkox/gggenes.

11. Feldgarden, M. *et al.* AMRFinderPlus and the Reference Gene Catalog facilitate examination of the genomic links among antimicrobial resistance, stress response, and virulence. *Sci. Rep.* **11**, 12728 (2021).
12. Camacho, C. *et al.* BLAST+: architecture and applications. *BMC Bioinformatics* **10**, 421 (2009).
13. Altschul, S. F., Gish, W., Miller, W., Myers, E. W. & Lipman, D. J. Basic local alignment search tool. *J. Mol. Biol.* **215**, 403–410 (1990).
