# Supplementary material for: A global genomic analysis of Salmonella Concord reveals lineages with high antimicrobial resistance in Ethiopia
Source: Nat Commun. 2023 Jun 14;14:3517. doi: 10.1038/s41467-023-38902-x (PMC10267216; doi:10.1038/s41467-023-38902-x)
Supplement: Supplementary file 3 — Description to Additional Supplementary Information [file 41467_2023_38902_MOESM3_ESM.pdf]

<u>Description of Additional Supplementary Files</u>

File Name: **Supplementary Data 1**

Description: Metadata and genomic data of all *S*. Concord isolates included in this study. The first six columns display isolate identifiers and accession numbers for both short- and long-read sequencing data. The subsequent four columns display sequence type (ST) and HC clusters determined using Enterobase. Columns 11 to 13 depict the consensus lineage and the original BAPS_level designations. The subsequent columns show whether an isolate passed sequencing quality control and whether it was considered for the analyses. The contextual metadata is displayed in columns 16 to 22, which includes laboratory-determined serotype, year of isolation, isolation source, institute that owns the isolate, travel history of a patient, and the orphanage in Addis Ababa associated with an isolate. The remaining columns provide information on genomic antimicrobial resistance (AMR) presence or absence, categorically and by gene, as well as the presence or absence of plasmid replicon genes.

File Name: **Supplementary Data 2**

Description: Overview of the Sensititre antimicrobial susceptibility testing results for a subset of 56 *S*. Concord isolates. The minimum inhibitory concentration (MIC) is provided for each isolate. For each antimicrobial or combination agent, the measured ranges and cutoffs are reported. Additionally, resistance combinations are reported, with the abbreviations MDR indicating multidrug resistance, XDR indicating extensive drug resistance, and PDR indicating pandrug resistance.
