# Supplementary material for: A global genomic analysis of Salmonella Concord reveals lineages with high antimicrobial resistance in Ethiopia
Source: Nat Commun. 2023 Jun 14;14:3517. doi: 10.1038/s41467-023-38902-x (PMC10267216; doi:10.1038/s41467-023-38902-x)
Supplement: Supplementary file 6 — Reporting Summary [file 41467_2023_38902_MOESM6_ESM.pdf]

nature portfolio

Corresponding author(s): Sandra Van Puyvelde, Wim L. Cuypers

Last updated by author(s): Apr 29, 2023

Reporting Summary

Nature Portfolio wishes to improve the reproducibility of the work that we publish. This form provides structure for consistency and transparency in reporting. For further information on Nature Portfolio policies, see our Editorial Policies and the Editorial Policy Checklist.

Statistics

For all statistical analyses, confirm that the following items are present in the figure legend, table legend, main text, or Methods section.

n/a	Confirmed	
☐	☒	The exact sample size (*n*) for each experimental group/condition, given as a discrete number and unit of measurement
☐	☒	A statement on whether measurements were taken from distinct samples or whether the same sample was measured repeatedly
☐	☒	The statistical test(s) used AND whether they are one- or two-sided<br>*Only common tests should be described solely by name; describe more complex techniques in the Methods section.*
☒	☐	A description of all covariates tested
☒	☐	A description of any assumptions or corrections, such as tests of normality and adjustment for multiple comparisons
☒	☐	A full description of the statistical parameters including central tendency (e.g. means) or other basic estimates (e.g. regression coefficient) AND variation (e.g. standard deviation) or associated estimates of uncertainty (e.g. confidence intervals)
☒	☐	For null hypothesis testing, the test statistic (e.g. *F*, *t*, *r*) with confidence intervals, effect sizes, degrees of freedom and *P* value noted<br>*Give P values as exact values whenever suitable.*
☒	☐	For Bayesian analysis, information on the choice of priors and Markov chain Monte Carlo settings
☒	☐	For hierarchical and complex designs, identification of the appropriate level for tests and full reporting of outcomes
☒	☐	Estimates of effect sizes (e.g. Cohen's *d*, Pearson's *r*), indicating how they were calculated

Our web collection on statistics for biologists contains articles on many of the points above.

Software and code

Policy information about availability of computer code

Data collection	Microsoft Excel for Microsoft 365 MSO (Version 2301 Build 16.0.16026.20002) 64 bit
Data analysis	Abricate v0.9.9<br>AMRFinder v3.10.30<br>Canu v2.1.1<br>Circlator v1.5.5<br>ComplexHeatmap v2.8.0<br>Fastbaps v1.0.7<br>FastQC v0.11.9<br>filtlong v0.2.0<br>Flye v2.8.2<br>genoplotR v0.8.11<br>gggenes v0.3.1<br>Ggtree v2.2.4<br>Gubbins v3.2.1<br>Guppy (ONT) v4.4.0<br>HGAP v3.0<br>Inkscape v0.92<br>Kraken v2.0.8-beta<br>Mauve

Mauve v2.4.1
Medaka v1.2.2
Medaka v1.2.2
minimap v2.17-r941
Mosdepth v0.3.1
MultiQC v1.8
Pilon v1.23
PRANK v.170427
Prokka v1.14.6
PycoQC v2.5.2
Quast v5.0.2
Quiver (smrtanalysis v2.3.0)
Raven v1.3.0
RAxML-NG v0.9.0
Redbean v2.5
Roary v3.13.0
samclip v0.3.0
samtools v1.9
SeqSero2 v1.2.1
smrtanalysis v2.3.0
snippy v4.6.0
snp-dists v0.7.0
snp-sites v2.5.1
Spades v3.15.5
Trimmomatic v0.39
Trycycler v0.4.1
UpSetR v1.4.0

For manuscripts utilizing custom algorithms or software that are central to the research but not yet described in published literature, software must be made available to editors and reviewers. We strongly encourage code deposition in a community repository (e.g. GitHub). See the Nature Portfolio guidelines for submitting code & software for further information.

Data

Policy information about availability of data

All manuscripts must include a data availability statement. This statement should provide the following information, where applicable:

- Accession codes, unique identifiers, or web links for publicly available datasets
- A description of any restrictions on data availability
- For clinical datasets or third party data, please ensure that the statement adheres to our policy

The sequencing reads generated using Illumina, Nanopore, and PacBio technologies can be accessed through ENA/SRA, and the corresponding accessions for each isolate can be found in Supplementary Data 1. Furthermore, assembled short-read data can be obtained from EnteroBase (https://enterobase.warwick.ac.uk/species/senterica/search_strains?query=workspace:79416). The data underlying Figure 1 are accessible via Enterobase (https://enterobase.warwick.ac.uk/species/senterica/search_strains?query=workspace:79432), while the data underlying Figure 2, including metadata, AMR genes, and replicon genes, are available through Supplementary Data 1. Antimicrobial susceptibility testing data, which are the basis of Supplementary Figures 15 and 16, and Supplementary Table 2, can be obtained from Supplementary Data 2. The PlasmidFinder database (retrieved on March 1, 2020) is available at https://cge.food.dtu.dk/services/PlasmidFinder/, while the AMRFinder database (used database version: 2022-05-26.1) is accessible via https://www.ncbi.nlm.nih.gov/pathogens/antimicrobial-resistance/AMRFinder/. Enterobase was searched on 21/07/2022 for all isolates included in HC_2000, and the search result can be accessed at https://enterobase.warwick.ac.uk/species/senterica/search_strains?query=workspace:79432. This paper includes source data.

Human research participants

Policy information about studies involving human research participants and Sex and Gender in Research.

Reporting on sex and gender	No human research participant data was collected.
Population characteristics	NA
Recruitment	NA
Ethics oversight	NA

Note that full information on the approval of the study protocol must also be provided in the manuscript.

Field-specific reporting

Please select the one below that is the best fit for your research. If you are not sure, read the appropriate sections before making your selection.

☐ Life sciences ☐ Behavioural & social sciences ☒ Ecological, evolutionary & environmental sciences

Ecological, evolutionary & environmental sciences study design

All studies must disclose on these points even when the disclosure is negative.

Study description	This study describes the population structure, antimicrobial resistance and genomic characteristics of 284 S. Concord isolates from Ethiopia and multiple other countries. Quantitative antimicrobial susceptibility testing data was generated for a subset of 56 isolates that were selected to represent different genomic AMR profiles and lineages.
Research sample	Salmonella enterica subspecies enterica serovar Concord (S. Concord) from Ethiopia and multiple other countries. Given the lack of comprehensive genome-wide information pertaining to S. Concord prior to our study, our selection criteria involved gathering all available isolates and genome sequences to construct a dataset that best represents the entirety of the S. Concord population.
Sampling strategy	We brought together S. Concord isolates and sequencing data from different collections including the collection of Institut Pasteur, DTU Denmark, The UK Health Security Agency (UKHSA), Jimma University, US Centers for Disease Control and Prevention (CDC), the Institute of Tropical Medicine Antwerp, Sciensano Belgium and The Center for Food Safety and Applied Nutrition (CFSAN) of the Food and Drug Administration (FDA).<br><br>All stored isolates that could be cultured were included for short-read sequencing. Publicly available short-read sequencing data of sufficient quality were were included.<br><br>Long-read sequencing data was generated using the Nanopore MinIon platform for ten isolates that represented different lineages and antimicrobial resistance profiles.<br><br>Antimicrobial susceptibility testing was performed of a subset of 56 isolates representative for different lineages and genomic AMR profiles. Since we observed (i) high similarity in genomic AMR profiles between isolates, and (ii) a high concordance between genotypic and phenotypic AMR in S. Concord (Supplementary Table 2), the sample size was sufficient.
Data collection	In total, 184 isolates were sequenced as part of this study, and details on cultivation and sequencing were provided in the manuscript. These isolates originated from the following collections, and the isolate collection was detailed previously in the listed publications:<br>- Collection of Institut Pasteur (historical contextual isolates + Fabré et al. 2009)<br>- DTU Denmark (Hendriksen et al. 2009, Hendriksen et al. 2010)<br>- Jimma University (Beyene et al. 2011)<br>- ScienSano Belgium (Vanhoof, 2011)<br><br>For Illumina data of the 126 isolates that were sequenced at other institutions, mostly within the context of ongoing surveillance programs, the isolates from UKHSA were sequenced as described in Chattaway et al. (2019), seven isolates from Institut Pasteur (lab ids: 156K, 202109373, 202101195, 201804751, 202205355, 202001598, 202005029) were sequenced as described in Jones et al. (2019) and isolates from the CDC and FDA collections were sequenced as part of the PulseNet surveillance system.<br><br>Metadata were provided as spreadsheets by the different isolate owners (from the institutions outlined above), and were combined using Microsoft Excel by W.L.C.
Timing and spatial scale	The year of isolation was available for 95.8% (272/284) isolates and ranged from 1944 to 2022. Fifty-percent of the isolates had been collected between 2006 and 2017.<br><br>The isolates were recovered in 12 different countries, including France (n = 116), UK (n = 53), USA (n = 39), Belgium (n = 17), Ethiopia (n = 27), Austria (n = 9), The Netherlands (n = 4), Denmark (n = 4), Turkey (n = 4), Czechia (n = 2), Ireland (n = 2), and Israel (n = 2). For five isolates the country of isolation was unknown.<br><br>The geographic origin was available for 45.5% (129/284) of the isolates. Most isolates were linked to Ethiopia (n = 101). These isolates originated from Ethiopian adoptees, were isolated from patients in Addis Ababa, or were linked to travel to Ethiopia. For 28 other isolates the travel history of a patient or the exact origin of the isolate was known: UK (n = 4), Turkey (n = 4), Kenya (n = 3), Zambia (n = 2), Thailand (n = 2), Israel (n = 2), Belize (n = 1), Central Africa (n = 1), Djibouti (n = 1), France (n = 1), Madagascar (n = 1), Saudi Arabia (n = 1), South Africa (n = 1), Tanzania (n = 1), USA (n = 1). One patient reported travel to the USA, Brazil and Tanzania, and one patient reported travel to South Africa, Zambia and Malawi (n = 1).<br><br>In order to provide a comprehensive analysis of the S. Concord population, this study aimed to retrospectively gather and evaluate all available isolates and genome sequences of S. Concord. To ensure a thorough representation of the entire population, the sampling timeline was selected to span from the first documented S. Concord isolate in 1944 up until 2022, which encompassed the most recent S. Concord sequences available at the time of analysing the data.
Data exclusions	Isolates that were unable to be resuscitated were excluded, as well as genome sequences that did not meet the quality criteria detailed in the manuscript. Only patient-unique isolates were considered.
Reproducibility	There was a high concordance between genotypic and phenotypic AMR for MDR, XDR and PDR. Antimicrobial susceptibility testing (AST) was performed only once using the Sensititre system with two custom panels in accordance with standard clinical and public health microbiology procedures.<br><br>Bioinformatics analyses were described in sufficient detail to reproduce the findings with the publicly available sequence data. Pylogenomics analyses for S. Concord super-lineage A were carried out thrice using different reference genomes as outgroups.

Randomization	Randomization was not required for the isolate selection process since (i) our goal was to collect all available S. Concord isolates and genome sequences to construct a dataset that represents the S. Concord population to date, and (ii) isolates were not allocated to different experimental groups.
Blinding	During data acquisition and analysis we worked with the isolate identifiers or accession codes to remove any potential for bias that might have arisen from knowledge of the specific samples being analyzed.

Did the study involve field work? ☐ Yes ☒ No

Reporting for specific materials, systems and methods

We require information from authors about some types of materials, experimental systems and methods used in many studies. Here, indicate whether each material, system or method listed is relevant to your study. If you are not sure if a list item applies to your research, read the appropriate section before selecting a response.

Materials & experimental systems

n/a	Involved in the study
☒	☐ Antibodies
☒	☐ Eukaryotic cell lines
☒	☐ Palaeontology and archaeology
☒	☐ Animals and other organisms
☒	☐ Clinical data
☒	☐ Dual use research of concern

Methods

n/a	Involved in the study
☒	☐ ChIP-seq
☒	☐ Flow cytometry
☒	☐ MRI-based neuroimaging

March 2021
